# Studies of chitosan-Prussian blue nanozyme in auditory protection: from cellular mechanisms to *in vivo* validation

**DOI:** 10.3389/fimmu.2026.1758392

**Published:** 2026-04-21

**Authors:** Yong Li, Na Wang, Pengcheng Ren, Yueyi Zhai, Peng Xie, Chunhuan Zhang, Yan Qu

**Affiliations:** 1 Department of Otolaryngology, Hebei Medical University, Shijiazhuang, China; 2 Department of Otolaryngology, Hebei General Hospital, Shijiazhuang, China; 3 Department of Otolaryngology, North China University of Science and Technology Affiliate Hospital, Tangshan, Hebei, China; 4 Department of Joint Surgery, Beijing Shijitan Hospital, Capital Medical University, Beijing, China; 5 Animal Laboratory, The Third Hospital of Hebei Medical University, Shijiazhuang, China; 6 The Third Hospital of Hebei Medical University, Shijiazhuang, China; 7 Department of Otolaryngology, The Third Hospital of Hebei Medical University, Shijiazhuang, China

**Keywords:** auditory protection, chitosan-Prussian blue nanozyme, inflammation, oxidative stress, toll-like receptor 4/nuclear factor-κB pathway

## Abstract

Oxidative stress and inflammation are interconnected drivers of cellular damage in pathologies ranging from neurodegenerative disorders to sensorineural hearing loss. We aimed to develop a chitosan-Prussian blue nanozyme (CS-PB) to target these processes in sensorineural hearing loss. CS-PB (35 μg/mL) pretreatment of H_2_O_2_-injured HEI-OC1 cochlear cells for 4 h markedly decreased the levels of reactive oxygen species (ROS) inside cells (from 3.8 to 2.4 relative fluorescence intensity) by approximately 37% (*P* < 0.001)-and concomitantly significantly decreased expression of the oxidative damage markers 4-HNE and 3-NT (both *P* < 0.001). The rate of apoptosis decreased from 27.5% in the H_2_O_2_-treated group to 14.1% following CS-PB treatment (P < 0.01). This reduction was accompanied by a significant downregulation of the pro-apoptotic proteins Bax (P < 0.05) and Cleaved-caspase-3 (P < 0.001), as well as an upregulation of the anti-apoptotic protein Bcl-XL (P < 0.01).Western blot analysis confirmed that CS–PB significantly downregulated TLR4 expression and inhibited downstream phosphorylation of p-P65/P65, while upregulating p-IκBα/IκBα protein (all *P* < 0.01) associated with reduction in the production of inflammatory cytokines like TNF-α, IL-1β, and IL-6. Treatment with the specific TLR4 antagonist TAK-242 (2 μM) mimicked the effect of CS-PB, with coadministration showing no additional ant-inflammatory effect (although HEI-OC1 viability was additionally increased), indicating that the anti-inflammatory properties of CS-PB were facilitated via the TLR4/NF-κB signaling pathway. *In vivo*, CS-PB pretreatment (2 mg/mL, 2 μL) was administered by carefully applying the solution to the round window membrane (RWM) using a microsyringe in a noise-induced hearing loss rat model, which significantly curtailed the noise-induced activation of the TLR4/NF-κB pathway in the cochlea. After 24 hours, the levels of p-P65/P65 and TLR4 decreased by 54% and 50%, respectively. (*P* < 0.001), and levels of the inflammatory cytokines IL-6, TNF-α and IL-1β were decreased by 53%, 48%, and 51% (*P* < 0.001). Furthermore, the degree of pathway inhibition showed a strong correlation with cytokine reduction (r = 0.87–0.89, all *P* < 0.001). CS-PB showed no significant cytotoxicity *in vitro* or *in vivo*, suggesting that its combined ROS-scavenging and TLR4/NF-κB–modulating properties may represent a potential therapeutic strategy, pending further mechanistic and translational validation.

## 1 Introduction

Oxidative stress and chronic inflammation are pivotal pathological drivers of cellular damage in pathologies ranging from neurodegenerative disorders to auditory hair cell damage, operating through a self-reinforcing vicious cycle. An overabundance of reactive oxygen species (ROS) disturbs redox balance, worsens mitochondrial dysfunction, and triggers pro-inflammatory pathways like the Toll-like receptor 4/nuclear factor-κB (TLR4/NF-κB) signaling. This irregular activation increases the release of cytokines such as interleukin-6 (IL-6),interleukin-1β (IL-1β) and tumor necrosis factor-α (TNF-α), thereby promoting neuronal apoptosis and contributing decisively to sensory cell injury and noise-induced hearing loss (NIHL) (1, 2). The murine cochlear cell line HEI-OC1 has been widely used in studies of otoprotective mechanisms (3) because it retains key properties of primary auditory hair cells, such as ROS sensitivity and TLR4 expression. TAK-242 (resatorvid), is a selective TLR4 antagonist, which binds to the intracellular cysteine 747 TLR4 residue, thereby blocking its interaction with adaptor proteins such as MyD88 and preventing downstream NF-κB activation (4–6). The connection between oxidative stress and apoptosis is well-established, particularly through the Bax/Cleaved-caspase-3 pathway (7–9). The Bax protein, part of the pro-apoptotic Bcl-2 family, is vital in triggering the release of cytochrome c from the mitochondria, subsequently activating caspase-3 (10). Despite the recognized importance of oxidative stress and inflammation in auditory pathology, conventional antioxidants and anti-inflammatory drugs commonly suffer from low bioavailability, poor specificity, and limited efficacy in targeting both pathological drivers. These challenges highlight the requirement for new therapeutic methods that can both neutralize ROS and inhibit inflammatory pathways to ensure effective otoprotection.

Recent advances in nanozyme technology have introduced Prussian blue analogues (PBAs) as promising ROS scavengers due to their enzyme-mimetic activities, biocompatibility, and stability under physiological conditions (11). For instance, FeCo-PBA nanoparticles (NPs) demonstrated robust ROS detoxification without cytotoxicity, enhancing cellular resilience in yeast fermentation models (11). Numerous previous studies have demonstrated that improvements in structural accuracy enhance the versatility of PBAs: Molybdenum-doped nanocages boost peroxidase/laccase activity for ultrasensitive biosensing (12). Therapeutically, mesoporous PBAs activate Nrf2 signaling to rescue cardiomyocytes from doxorubicin toxicity (13), while Prussian blue nanoparticles (PBNPs) block osteoclastogenesis via ROS-NF-κB/MAPK axis interception (14), demonstrating dual ROS clearance and pathway regulation unreported in metal-organic frameworks. Diagnostically, CoFe PBAs measure H_2_O_2_/dopamine through peroxidase-mediated chromogenic reactions (15), more rapidly than ELISA (15 vs.180 mins). Meanwhile, superoxide dismutase (SOD)-mimetic PBAs reverse myocardial ischemia-reperfusion injury by neutralizing superoxide radicals (16), reducing infarct by 41% compared to controls. These advances establish PBAs as redox architects that orchestrate oxidative homeostasis through structure-activity relationships, propelling their translation from laboratories to clinics. However, their application in neuroinflammatory and otoprotective contexts remains underexplored.

Chitosan, the N-deacetylated derivative of chitin, is distinguished as the only alkaline polysaccharide among naturally occurring sugars. Structurally reactive free amino groups, endow it with unique features including biodegradability, cell affinity, and suceptibility to chemical modification enabling diverse biological functions (2). Chitosan offers significant benefits as a drug carrier, including stabilization of pharmaceutical components, improved drug absorption, and controlled dissolution rates enabling targeted organ delivery and protection against gastric irritation. Chitosan is commonly used in targeted delivery systems, especially as nanoparticles and microspheres (1). Chitosan has emerged as a multifunctional biomaterial with intrinsic antioxidant and anti-inflammatory properties (1, 11). Chitosan has also been reported to modulate immune responses via suppression of the TLR4/NF-κB pathway (2), and to enhance the pharmacokinetic behavior of drugs through improved absorption and controlled release (11). These characteristics make chitosan an ideal matrix for the construction of hybrid nanocomposites.

Integrating chitosan with Prussian blue nanozymes represents a compelling strategy to exploit their complementary functionalities: attenuation of TLR4/NF-κB activation (2), combined with catalase (CAT)- and SOD-like activities (11), offering dual modulation of oxidative and inflammatory pathways. Despite progress in nanozyme applications, the therapeutic potential of chitosan-Prussian blue hybrids in auditory cells remains unexplored.

In this study, we sought to investigate the application of a novel nanotherapeutic—chitosan-Prussian blue nanozyme (CS-PB) to investigate a potential dual protective role against auditory cell damage. We hypothesized that CS-PB would (1) scavenge ROS through the intrinsic SOD- and CAT-like activities of Prussian blue, and (2) suppress inflammation via chitosan-mediated inhibition of the Toll-like receptor 4/nuclear factor-κB (TLR4/NF-κB) pathway; serving to preserve the integrity of HEI-OC1 auditory cells under oxidative stress.

Recognizing the limitations of an *in vitro* model, which cannot fully replicate the intricate cochlear microenvironment or predict therapeutic performance in living systems, we aimed to establish a noise-induced hearing loss (NIHL) model in Sprague-Dawley rats,. seeking to (1) validate the auditory protective effects of CS-PB through auditory brainstem response (ABR) and cochlear tissue analysis, (2) confirm the *in vivo* relevance of the dual mechanisms of ROS scavenging + TLR4/NF-κB inhibition, and (3) to assess the biosafety of local CS-PB administration via round window membrane delivery. The long-term aim was to delineate the interplay between oxidative stress and inflammation in auditory injury to provide a foundation for translational development of CS-PB as a potential nanotherapeutic strategy for hearing loss and possibly related sensory disorders.

## 2 Materials and methods

### 2.1 Preparation, physicochemical characterization, and activity testing of CS-PB nanozyme

#### 2.1.1 Preparation of CS–ascorbate transparent colloid

Sodium ascorbate (440.4 mg) was dissolved in 50 mL ultrapure water under magnetic stirring until fully dissolved. Chitosan (500 mg) was then gradually added and stirred for 30 min. The pH was adjusted to 4.5 using dilute hydrochloric acid. The mixture was heated and stirred in a 60 °C water bath for 2 h to obtain a completely transparent CS–ascorbate colloid. After cooling to room temperature, the colloid was used for subsequent synthesis.

#### 2.1.2 Visible-light-assisted synthesis of CS-PB

Potassium ferricyanide (16.46 mg) was dissolved in 50 mL ultrapure water to prepare a 1 mM K3_33[Fe(CN)6] solution. This solution was slowly poured into the 50 mL CS–ascorbate colloid under stirring, yielding a pale-yellow mixture. The mixture was then irradiated using a 30 W white LED light source (distance 5–10 cm) for 3 min with continuous stirring. The reaction was stopped immediately when the solution turned deep blue, indicating the formation of CS-PB nanozyme.

The suspension was transferred into centrifuge tubes and centrifuged at 7000 rpm for 3 min; the supernatant was discarded. The pellet was resuspended in 20 mL ultrapure water by vortexing and centrifuged at 10,000 rpm for 5 min. This washing step was repeated three times. The final pellet was freeze-dried at -50 °C under vacuum (<10 Pa) for 12–24 h to obtain CS-PB nanozyme powder (**Supplementary Figure 1**). The physicochemical characterization and activity testing methods for CS-PB are detailed in the **Supplementary Materials**.

### 2.2 Antioxidant activity assays

#### 2.2.1 DPPH radical scavenging assay

DPPH ethanol working solution (0.2 mM) was prepared by dissolving 8 mg DPPH in 100 mL absolute ethanol, protected from light and sonicated for 15 min. In a 96-well plate, 5 μL CS-PB at various concentrations was mixed with 195 μL DPPH solution (sample group). A sample blank (5 μL sample + 195 μL ethanol) and a control (5 μL water + 195 μL DPPH) were included. After incubation at 37 °C for 30 min in the dark, absorbance was measured at 517 nm. Scavenging (%) was calculated as:

DPPH scavenging (%) = [1−(As−Ab)/Ac] × 100%.

where As is sample group absorbance, Ab is sample blank absorbance, and Ac is control absorbance.

#### 2.2.2 ABTS radical scavenging assay

ABTS·+ stock solution was prepared by mixing equal volumes of 7 mM ABTS diammonium salt and 2.45 mM K2S2O8, incubated in the dark for 12–16 h at room temperature. The ABTS·+ working solution was obtained by diluting the stock with ethanol to A734 = 0.700 ± 0.020. In a 96-well plate, 10 μL CS-PB was mixed with 190 μL ABTS working solution (sample group). A sample blank (10 μL sample + 190 μL ethanol) and a control (10 μL water + 190 μL ABTS working solution) were prepared. After standing at 37 °C for 6 min in the dark, absorbance was measured at 734 nm. Scavenging (%) was calculated similarly to the DPPH assay.

### 2.3 Experimental animals and drug administration through the round window membrane

An established NIHL model in Sprague–Dawley rats was employed, where 120 dB sound pressure level (SPL) broadband noise exposure (12 hour (h)/day for three consecutive days) reliably induces cochlear damage resembling the clinical condition, including outer hair cell loss and activation of the TLR4/NF-κB pathway.

A group of 70 male Sprague-Dawley rats, aged between 2 and 3 months and weighing 200–250 grams, were obtained from Beijing HFK Bioscience Co., Ltd. The rats were kept in an SPF animal facility where the temperature was maintained at 22-24 °C, humidity at 55 ± 5%, and a 12-hour light/dark cycle, with unlimited access to food and water. Prior to studies, the Preyer reflex was tested by delivering a 60 dB SPL click stimulus, of 10 milliseconds (ms) duration, to each ear; rats with no visible pinna reflex were excluded. The protocol for the experiment received approval from the Animal Ethics Committee of the Third Hospital of Hebei Medical University. The rats were assigned to three groups at random. with sample sizes and grouping details as follows: Control group: Ten rats were included with no noise exposure. A total of 2 μL normal saline was administered by carefully applying the solution to the round window membrane (RWM) using a microsyringe. Noise-Vehicle group: Thirty rats were randomly divided into three time-point subgroups (n = 10 per subgroup) based on the interval after noise exposure: 6 h, 24 h, and 72 h post-noise. All rats in this group were exposed to broadband noise for 12 h/day over three consecutive days. Two microliters of normal saline were administered via RWM by carefully applying the solution with a microsyringe 1 day prior to noise exposure. Noise-CS-PB group: Thirty rats were also subdivided into the same three time-point subgroups (n = 10 per subgroup) and subjected to the identical noise exposure protocol as the Noise-Vehicle group. Two microliters of chitosan-Prussian blue nanozyme (CS-PB, 2 mg/mL; obtained from Zhengzhou Yanben Biotechnology Co., Ltd.) was administered by carefully applying the solution to the RWM using a microsyringe 1 day before noise exposure. This concentration was selected for subsequent experiments based on preliminary pilot observations indicating relatively favorable protective trends.

The rats were anesthetized with an intraperitoneal injection of sodium pentobarbital at a dose of 40 mg/kg and maintained at 37 °C on a temperature-controlled table. A 1.5 cm retro-auricular incision exposed the temporal bone, and a 0.5–1 mm bullastomy was drilled under a 20–40× operating microscope to visualize the RWM. A 25 μL microsyringe (33-gauge needle) delivered 2 μL of test agents (1/2/4 mg/mL CS-PB) or vehicle (normal saline) onto the RWM: the needle tip was positioned 1–2 mm above the membrane, with slow injection (0.5 μL/s) to ensure full coverage, without overflow. Medical bone wax was used to seal the bullastomy, and the incision was sutured with 4–0 polyglactin 910. Postoperatively, rats recovered in a warm chamber and were monitored daily for 3 days to assess general condition and exclude complications. This RWM application method has been previously validated in our laboratory to achieve effective cochlear delivery in a rat model of noise-induced hearing loss (17).

### 2.4 Pilot study for screening the exploratory concentration of CS-PB

#### 2.4.1 Experimental design and groupings

A preliminary pilot experiment was conducted to obtain exploratory information for selecting a working concentration of CS-PB for subsequent *in vivo* experiments. Four adult Sprague–Dawley rats (eight ears) were included in this pilot study, with each ear considered an independent experimental unit. The animals were randomly assigned to four groups according to a random number table:

Vehicle control (blank) group: 2 μL of normal saline was administered via a single preventive intratympanic injection.

CS-PB 1 mg/mL group: 2 μL of 1 mg/mL CS-PB was administered the above method.

CS-PB 2 mg/mL group: 2 μL of 2 mg/mL CS-PB was administered using the above method.

CS-PB 4 mg/mL group: 2 μL of 4 mg/mL CS-PB was administered using the above method.

This pilot experiment was designed to observe general trends in hearing outcomes across different concentrations, rather than to establish a statistically optimized dose.

#### 2.4.2 Noise exposure

Rats were subjected to constant broadband white noise a day following surgery. (120–125 dB SPL in a custom-fabricated sound-attenuating chamber, with daily exposure lasting 12 h on three successive days. The noise delivery system comprised an AWA61290M dual-channel acoustic analyzer and an AWA5870B 100W power amplifier to ensure stable noise output. Control rats were housed in the identical chamber under silent conditions for the same duration as the noise-exposed groups. Cross-interference from sound pressure between adjacent animals, was eliminated by separating individuals using wire mesh partitions within the chamber. To maintain their normal physiological state, rats in the sound chamber were given unrestricted access to food and water.

#### 2.4.3 ABR recording

In all rats, ABRs were recorded in a soundproof chamber at 1, 3, 7, and 14 days after noise exposure. Tone burst stimuli were generated across an intensity range of 20–100 dB SPL, with 5 dB stepwise reductions. The stimuli had a rise and fall time of 0.2 ms, a plateau lasting 1 ms, and were presented at frequencies of 4, 8, 16, 24, and 32 kHz. Amplitude modulation was accomplished with a sound generator, a real-time attenuation processor, and a programmable attenuator from Tucker-Davis Technology in Alachua, FL, USA. ABR thresholds were defined as the minimum stimulus intensity (in dB SPL) capable of eliciting a reproducible waveform onset.

#### 2.4.4 Statistical analysis of the pilot study

The ABR thresholds (dB SPL) at each frequency were analyzed using linear mixed-effects models (LMMs). The fixed effects were group (control group, 1 mg/mL, 2 mg/mL and 4 mg/mL subgroups) and time (Days 1, 3, 7, 14), with ear as a random intercept. The Holm method was used to correct for multiple comparisons (dose vs. control groups at each time point, and comparisons between different time points within each group). The estimated marginal means (EMMeans) of the model and 95% confidence intervals (CIs) were reported.

Considering the limited sample size (around 2 ears per group), statistical inference focused on effect sizes and confidence intervals, with *P*-values used as exploratory indicators. All analyses were performed separately by frequency. The visualization graphs showed the group average ± standard error of the mean (SEM) along with data points for each individual ear.

Due to the limited sample size of the pilot study, some model-derived estimates showed minimal variance; therefore, pilot data are presented primarily to illustrate trends rather than to support definitive statistical inference. Among the tested concentrations, 2 mg/mL CS-PB showed relatively consistent reductions in ABR thresholds across several tested frequencies, suggesting a potential protective effect against noise-induced hearing damage. Based on these exploratory observations, 2 mg/mL was selected as a working concentration for subsequent *in vivo* experiments. Because of the small sample size, the pilot data are presented primarily to illustrate preliminary trends and to guide experimental design, rather than to support definitive statistical conclusions.

### 2.5 Screening of TAK-242 experimental concentration and determination of cytotoxicity, and biosafety

HEI-OC1 cells were seeded into 96-well plates at a concentration of 1x10^4^ cells per well and categorized into the following groups: Normal control group: cells cultured with complete medium only. TAK-242-treated groups: cells subjected to different doses of TAK-242 (0.25, 0.5, 1, 2, 4 and 8 μM) in complete medium. After seeding, cells in both groups were incubated for 24, 48, and 72 h. Cell viability was assessed at each time interval using the Cell Counting Kit-8 (CCK-8) assay:10 μL of CCK-8 reagent was introduced to each well, and the plates were incubated at 37°C for 2 hours. Using a microplate reader, the optical density (OD) at 450 nm was measured, and cell viability was determined as follows:

Cell viability (%) = (OD value of TAK-242-treated group/OD value of normal control group) × 100%.

The ideal concentration of TAK-242 was identified based on the criteria of maintaining ≥ 80% cell viability (to ensure biosafety) and potential functional effectiveness. The corresponding pretreatment time was determined as the duration achieving stable cell viability within this concentration range. To further verify biosafety, cells were cultured with TAK-242 at the screened optimal concentration for 24, 48, and 72 h and cell viability was re-evaluated using the CCK-8 method to confirm consistent low cytotoxicity across time points.

### 2.6 Effect of TAK-242 and CS-PB on H_2_O_2_-induced cytotoxicity in HEI-OC1 cells

HEI-OC1 cells were divided into a normal control group, an H_2_O_2_- treated group, and groups treated with CS-PB, TAK-242 and TAK-242 + CS-PB. In the 96-well plates, cells were planted at a density of 1×10^4^ cells per well. Following H_2_O_2_ exposure, the medium was swapped out for fresh complete medium, and the cells were incubated for another 24 hours. To determine cell viability, the CCK-8 assay was employed: 10 μL of CCK-8 reagent was introduced to each well, then incubated at 37°C for 2 hours. A microplate reader was used to measure the OD values at 450 nm, and cell viability was determined as follows:

Cell viability (%) = (OD value of treatment group/OD value of normal control group) × 100%.

The cell viability results were used to compare the protective effects of individual and combined treatments against H_2_O_2_-induced cytotoxicity.

### 2.7 Determination of cellular mitochondrial ROS by flow cytometry

HEI-OC1 cells were planted in 6-well plates with a concentration of 1×10^6^ cells per well. After 24 h culture, cells were treated according to grouping. Following the treatment, the supernatant was taken out, and the cells were rinsed with phosphate-buffered saline (PBS). The 2’,7’-dichlorofluorescein diacetate (DCFH-DA) reagent was diluted at a ratio of 1:1000 using Dulbecco’s Modified Eagle Medium (DMEM), subsequently applied to the cells, and incubated at 37°C for a duration of 30 minutes. PBS was used to wash the cells after the staining reagent had been removed. After trypsin digestion and centrifugation, the cell pellet was resuspended and transferred to a flow-cytometry-specific tube for fluorescence measurement.

### 2.8 Protein extraction and Western blotting

Following treatment, HEI-OC1 cells from each group underwent protein extraction. Initially, the culture medium was aspirated, and the cells were washed twice with pre-cooled PBS at pH 7.4 to eliminate any residual medium. The cells were then gently scraped using a sterile cell scraper and transferred into 1.5 mL centrifuge tubes. Subsequently, the cell suspension was centrifuged at 1,000 g for 5 minutes at 4 °C. The supernatant was discarded to retain the cell pellet. The cell pellet underwent lysis with pre-chilled RIPA buffer, which was enhanced with 1% PMSF, using a volume of 100 μL for every 1×10^6^ cells. After a brief vortexing, the mixture was incubated on ice for 30 minutes, with gentle shaking every 10 minutes. The sample was then centrifuged at 12,000 g for 30 minutes at 4 °C. The supernatant obtained, which represents the total protein extract, was meticulously moved to a new tube and kept at -80°C for future analysis. The protein concentration was quantified utilizing a bicinchoninic acid (BCA) Protein Assay Kit (Beyotime, China). This involved the addition of 20 μL of either a standard protein solution, comprising 5 mg/mL bovine serum albumin (BSA), or a diluted sample into a 96-well plate in triplicate. Subsequently, 200 μL of BCA working solution was added, and the mixture was incubated at 37 °C for 30 minutes. The optical density (OD) was measured at 562 nm using a microplate reader. The protein concentration was then calculated based on a standard curve, and samples were adjusted to equal concentrations using a lysis buffer. For SDS-PAGE and Western blot analysis, each 50 μg protein sample was denatured by mixing with five times its volume of SDS loading buffer and heating at 95°C for 10 minutes. The proteins were then resolved using a 10% SDS-PAGE, initially at 80 volts for 30 minutes to concentrate the gel, followed by 120 volts for 90 minutes to separate the proteins. The proteins were subsequently transferred onto polyvinylidene fluoride (PVDF) membranes (Millipore, USA) employing a wet transfer technique at a current of 300 mA for a duration of 90 minutes. This method is appropriate for proteins with molecular weights ranging from 20 to 100 kDa and utilizes a transfer buffer comprising 20% methanol. Following the transfer, the membranes were incubated in a blocking solution of 5% non-fat milk in tris-buffered saline with Polysorbate 20 (TBST) at ambient temperature for 2 hours. They were then incubated overnight at 4 °C with primary antibodies, including anti-Bcl-XL (1:1,000, CST, #2764), anti-Bax (1:1,000, CST, #5023), anti-TNF-α (1:1,000, Abcam, ab6671), anti-IL-1β (1:1,000, Abcam, ab9722), anti-TLR4 (1:1,000, CST, #14358), anti-p-NF-κB p65 (1:1,000, CST, #3033), anti-NF-κB p65 (1:1,000, CST, #8242), and anti-β-actin (1:5,000, Servicebio, GB12001 as an internal reference), all diluted in 5% BSA/TBST. Following three washes with TBST (10 minutes each), the samples were incubated at room temperature for one hour with a horseradish peroxidase (HRP)-conjugated goat anti-rabbit secondary antibody (dilution 1:5,000, Servicebio, GB23303). Subsequently, the samples were visualized using an enhanced chemiluminescence (ECL) detection kit (Millipore, USA). The imaging process was conducted using a Bio-Rad ChemiDoc XRS+ system, and band intensities were measured with ImageJ software (NIH, USA); the relative expression was determined by comparing the target protein band intensity to that of β-actin. To ensure reliability, all experiments were conducted three times using independent cell cultures.

### 2.9 Immunofluorescence staining and laser confocal microscopy imaging

The HEI-OC1 cells subjected to treatment were fixed using 4% paraformaldehyde for 15 minutes. This was followed by permeabilization with 0.5% Triton X-100 at room temperature for 20 minutes. Subsequently, the cells were blocked with 10% goat serum and incubated with the primary antibodies, 4-hydroxynon-2-enal (4-HNE) and 3-nitrotyrosine (3-NT), at 4 °C overnight. After washing with PBST, the cells were incubated with the secondary antibody in the dark at room temperature for 1 hour. The cells were then counterstained with 4’,6-diamidino-2-phenylindole (DAPI) and mounted using a mounting medium containing an anti-fluorescence quenching agent. The images were captured with the help of a laser confocal microscope.

### 2.10 Biosafety assessment of CS-PB in rats

Prior to sacrificing the aforementioned experimental rats, serum samples were taken for biochemical analyses, such as alanine aminotransferase (ALT), aspartate aminotransferase (AST), blood urea nitrogen (BUN), creatinine (CR), and creatine kinase (CK). Moreover, the major organs, which include the heart, brain, liver, spleen, and kidney, were obtained for pathological study following hematoxylin and eosin (H&E) staining.

### 2.11 Cochlear tissue processing for Western blotting and PCR

#### 2.11.1 Cochlear tissue collection and homogenization

After euthanasia, the cochleae were quickly dissected on ice and washed with ice-cold phosphate-buffered saline (PBS, pH 7.4) to remove any remaining blood and debris. Each cochlear sample was then placed into a pre-chilled glass homogenizer, and radioimmunoprecipitation assay (RIPA) lysis buffer (Solarbio, R0010), supplemented with 1% phenylmethylsulfonyl fluoride (PMSF) and 1% protease inhibitor cocktail (Solarbio, P6730), was added at a ratio of 100 μL per 10 mg of tissue. The tissue was homogenized on ice for 30 seconds, followed by a 10-second rest, for a total of 3 to 5 cycles until complete lysis was achieved. Subsequently, the homogenate was transferred to a 1.5 mL centrifuge tube and incubated on ice for 30 minutes, with gentle mixing every 10 minutes.

#### 2.11.2 Protein extraction and quantification for Western blotting

The lysate underwent centrifugation at 12,000 × g for 15 minutes at 4 °C, and the supernatant was gathered as the total protein extract. The concentration of protein was measured with a BCA Protein Assay Kit (Solarbio, PC0020) following the manufacturer’s instructions, using BSA as a reference.

After quantification, each protein sample was mixed with 5× loading buffer containing DTT (Solarbio, P1040) and boiled for 10 min in a dry bath at 95 °C. Denatured protein samples were briefly centrifuged, sealed, and stored at −80 °C until SDS-PAGE and Western blotting analysis.

#### 2.11.3 RNA extraction and cDNA synthesis for real-time PCR

In accordance with the manufacturer’s protocol, total RNA was extracted utilizing the TRNzol Universal Reagent (TIANGEN, DP424). Approximately 1 mL of TRNzol was employed for every 50–100 mg of cochlear tissue. Following homogenization, the samples were incubated at room temperature for 5 minutes to ensure complete lysis. Subsequently, 0.2 mL of chloroform was added per 1 mL of TRNzol. The mixture was then vigorously agitated for 15 seconds, incubated for 3 minutes, and centrifuged at 12,000 × g for 15 minutes at 4 °C. The upper aqueous phase was carefully transferred to a new tube and mixed with an equal volume of isopropanol, followed by a 10-minute incubation at room temperature to facilitate RNA precipitation. RNA was precipitated by centrifugation at 12,000 × g for 10 minutes at 4 °C. The resulting RNA pellet was washed twice with 75% ethanol, prepared with DEPC-treated water, and centrifuged at 7,500 × g for 5 minutes each wash. The RNA pellet was allowed to air-dry for 5 to 10 minutes before being dissolved in RNase-free water. The RNA purity and concentration were assessed utilizing a NanoDrop™ One spectrophotometer (Thermo Fisher Scientific, USA). In accordance with the manufacturer’s instructions, first-strand cDNA synthesis was performed using the PrimeScript™ RT Reagent Kit (TaKaRa, Japan). The synthesized cDNA was subsequently stored at −20 °C until required for Real-Time PCR analysis.

### 2.12 Statistical analysis

Data analysis and graph generation were conducted using Origin 9.0 and GraphPad Prism version 9.5.1. Initially, all measurement data underwent normality testing via the Shapiro-Wilk test and homogeneity of variance assessment using Levene’s test. For datasets adhering to normal distribution and homogeneity of variance, results were presented as mean ± standard deviation (SD). Comparisons among multiple groups were performed using one-way analysis of variance (ANOVA) followed by Tukey’s *post-hoc* test. In cases where the data did not satisfy these assumptions, the non-parametric Kruskal-Wallis H test was employed, with Dunn’s *post-hoc* test for subsequent analysis. Statistical significance was established at P < 0.05, with P < 0.01 indicating high significance and P < 0.001 denoting extreme significance.

## 3 Results

### 3.1 Physicochemical characterization of PB/CS-ACE nanozyme

#### 3.1.1 Particle size distribution, surface potential, and morphology analysis

The CS-PB nanoparticles exhibited an average hydrodynamic diameter of 42.1 ± 4.5 nm (**Supplementary Figure 2A**). The measured polydispersity index (PDI) of 0.21 ± 0.02 further suggests that the nanoparticles possess satisfactory dispersibility and moderate monodispersity in aqueous media. The zeta potential distribution also presented a well-defined single peak, with an average surface potential of +18.7 ± 2.1 mV (**Supplementary Figure 2B**). Low-magnification TEM images revealed that CS-PB presented a relatively uniform spherical morphology with good dispersion and without apparent large-scale aggregation, suggesting that the chitosan matrix contributes to stabilizing the Prussian blue nanoparticles during synthesis (**Supplementary Figures 3A, B**). At higher magnification, abundant nanoscale particles with clearly defined boundaries were observed, and the particle sizes appeared to be relatively concentrated, indicating the formation of stable nanostructures (**Supplementary Figures 3C–E**). High-resolution TEM (HRTEM) further displayed distinct lattice fringes, demonstrating good crystallinity of CS-PB and implying that the Prussian blue component retained its crystalline structure after integration with chitosan (**Supplementary Figure 3F**).

#### 3.1.2 Fourier transform infrared spectroscopy spectral analysis

FT-IR spectra (**Supplementary Figures 4A, B**) were used to examine the chemical structure of CS-PB and the interactions among its components, with CS-CND (chitosan–carbon dot composite) as the control. CS-PB exhibited absorption bands at 2916.81 cm^-1^(stretching vibration of saturated C–H), 1573.15 cm^-1^ (bending vibration of –NH2_22), and 1023.23/1062.22 cm^-1^ (C–O–C stretching of ether linkages in glucosamine units). These bands were consistent with the characteristic peaks of CS-CND (2876.47 cm^-1^, 1596.81 cm^-1^, and 1079.94 cm^-1^, indicating that the polysaccharide backbone and amino functionalities of chitosan were preserved after synthesis. A prominent band at 556.26 cm^-1^ was observed in CS-PB, which is attributable to the characteristic stretching vibration of the Fe–CN–Fe bridging structure in Prussian blue. This peak was absent in the CS-CND spectrum, confirming the presence of PB within the composite. In addition, CS-PB showed a weak band at 1713.44 cm^-1,^ which can be assigned to a shifted carbonyl-related vibration associated with coordination interactions between carboxyl groups and Fe species in PB. Moreover, a broadened band centered at 3263.59 cm^-1^ displayed higher intensity than that of CS-CND, consistent with enhanced hydrogen-bonding interactions involving hydroxyl groups (e.g., from ascorbate) within the CS-PB network. Collectively, the FT-IR results support the successful integration of PB and ascorbate into the chitosan-based composite.

#### 3.1.3 Electron spin resonance characterization of ROS-scavenging capacity

ESR spectroscopy was employed to evaluate the reactive oxygen species (ROS)-scavenging capability of CS-PB nanozyme. The control group exhibited a strong radical-associated ESR signal within the magnetic field range of 3460–3530 G, with a characteristic distribution at g≈2.03g \approx 2.03g≈2.03, indicating the presence of abundant paramagnetic radical species (**Supplementary Figure 5**). Upon addition of CS-PB, the ESR signal intensity markedly decreased while the overall spectral profile remained largely unchanged, suggesting a substantial reduction in radical concentration. Notably, signal attenuation was observed across the entire ggg-value region, supporting the broad ROS-quenching capacity of CS-PB rather than an effect restricted to a single radical type. To further quantify radical scavenging, the ESR signal intensity was integrated and presented as a bar plot. Compared with the control group, the CS-PB nanozyme group exhibited a markedly reduced integrated ESR signal, with an overall decrease of approximately 79% (**Supplementary Figure 6**).

#### 3.1.4 X-ray photoelectron spectroscopy analysis of CS-PB nanozyme

XPS was performed to analyze the surface elemental composition of the CS-PB nanozyme. The survey spectrum indicated that the sample surface was dominated by carbon, with additional signals corresponding to oxygen and iron. A strong peak at ~285 eV was assigned to C 1s, while the signal at ~530 eV corresponded to O 1s (**Supplementary Figure 7**). In addition, characteristic Fe 2p peaks were observed in the 700–730 eV region, confirming the successful incorporation of Fe species into the material. No evident impurity-related peaks were detected in the survey spectrum, supporting a relatively high surface purity of the prepared nanozyme. High-resolution C 1s spectra displayed a dominant peak centered at ~285 eV, which can be assigned to C–C/C–H bonds within the chitosan backbone. Peak broadening with a slight shoulder suggested the presence of oxygen- and nitrogen-containing functionalities (e.g., C–O and C–N), consistent with the polysaccharide-based organic matrix (**Supplementary Figure 8**). High-resolution Fe 2p spectra (**Supplementary Figure 9**) showed discernible Fe-related signals, further supporting the presence of Fe-containing active centers. The Fe 2p features were relatively weak and broadened, which may be attributed to the low surface abundance of Fe and partial attenuation by the chitosan coating layer. High-resolution XPS analysis of Fe 2p was further performed (**Supplementary Figure 10**). Two characteristic peaks at binding energies of 708.5 eV and 721.5 eV were assigned to Fe 2p3/2 and Fe 2p1/2, respectively, suggesting that Fe is predominantly present in the Fe2+ state and can be attributed to a [Fe^2+^ − CN] coordination environment.

#### 3.1.5 XRD pattern analysis of CS-PB nanozyme

To further elucidate the crystalline features of CS-PB nanozyme, X-ray diffraction (XRD) analysis was performed. Within the 2θ scanning range of 5–80°, multiple distinct diffraction peaks were observed in the low-angle region (~8–25°) (**Supplementary Figure 11**), indicating the presence of a certain degree of long-range ordering in the material. After background subtraction and smoothing (**Supplementary Figure 12**), the major reflections could be clearly identified at 2θ = 8.83°, 11.76°, 12.08°, 19.31°, and 22.96°. In addition, weaker yet discernible diffraction signals were still detectable at higher angles (35.59° and 40.43°).

### 3.2 Multi-enzyme mimetic activities and pH stability

#### 3.2.1 SOD-like activity

To further evaluate the multi-enzyme mimetic properties of CS-PB, its superoxide dismutase (SOD)-like activity was assessed using a xanthine/xanthine oxidase (XOD)–cytochrome c competitive inhibition assay. Results showed that increasing concentrations of CS-PB markedly reduced the reduction rate of cytochrome c at 550 nm, resulting in a concentration-dependent increase in inhibition (**Supplementary Figure 13**). These results indicate that CS-PB effectively scavenges superoxide anion radicals (·O2−). The IC_50_ value calculated from the inhibition curve further supports the SOD-like catalytic activity of CS-PB and its capability for ROS modulation.

#### 3.2.2 CAT-like activity

The catalase (CAT)-like activity of CS-PB nanozyme was evaluated using a dissolved oxygen probe. After the addition of H_2_O_2_, the dissolved oxygen level increased rapidly and linearly within the first 5 min (slope = 0.32 ± 0.02 mg/L·min), followed by a gradual plateau (**Supplementary Figure 14**), indicating efficient catalytic decomposition of H2O2 by the nanozyme.

#### 3.2.3 POD- and OXD-like activities

The peroxidase (POD)-like and oxidase (OXD)-like activities were assessed using the TMB oxidation assay (**Supplementary Figure 15**). With increasing CS-PB concentration, the absorbance signal showed a slight concentration-dependent increase; however, the overall change remained limited, indicating relatively low catalytic efficiency under the tested conditions. These results suggest that CS-PB exhibits only modest pro-oxidant catalytic activity and is primarily oriented toward antioxidant regulation rather than ROS amplification. The comparatively weak POD/OXD-like activity may be advantageous for inner-ear protection, as excessive pro-oxidant catalysis could exacerbate oxidative stress in cochlear tissues.

#### 3.2.4 CS-PB maintains stable enzyme-mimetic activity under physiologically relevant pH conditions

To further evaluate the enzyme-mimetic performance of CS-PB under physiologically relevant pH conditions, its SOD-like activity was quantitatively compared at pH 6.8 and pH 7.4. CS-PB maintained stable SOD-like catalytic activity at both pH values (**Supplementary Figure 16**), indicating that its enzyme-mimetic function is applicable under mildly acidic as well as physiological environments. At lower concentrations (< 0.01 mg/mL), the inhibition rate at pH 6.8 was slightly higher than that at pH 7.4, suggesting enhanced SOD-like activity under mildly acidic conditions, which is relevant to inflammatory microenvironments. At higher concentrations (> 0.05 mg/mL), the inhibition rates under the two pH conditions converged to a similar level (approximately 70%), indicating that the pH effect became less pronounced when the concentration-dependent effect dominated and that CS-PB retained high catalytic activity at physiological pH.

#### 3.2.5 DPPH and ABTS radical scavenging capacity

CS-PB exhibited strong, concentration-dependent antioxidant activity in both DPPH and ABTS assays (**Supplementary Figure 17**). At 50 μg/mL, CS-PB achieved 91.2 ± 2.3% DPPH scavenging (EC50 = 18.5 ± 1.2 μg/mL) and 88.7 ± 1.8% ABTS+scavenging (EC50 = 21.3 ± 1.5 μg/mL), with EC50 values comparable to vitamin C (15.2 ± 0.8 and 18.5 ± 1.0 μg/mL, respectively).

### 3.3 Effect of CS-PB on H_2_O_2_-induced oxidative injury and inflammation in HEI-OC1 auditory cells

#### 3.3.1 Construction of H_2_O_2_-induced HEI-OC1 oxidative stress model and evaluation of CS-PB pretreatment

**Figure 1:**
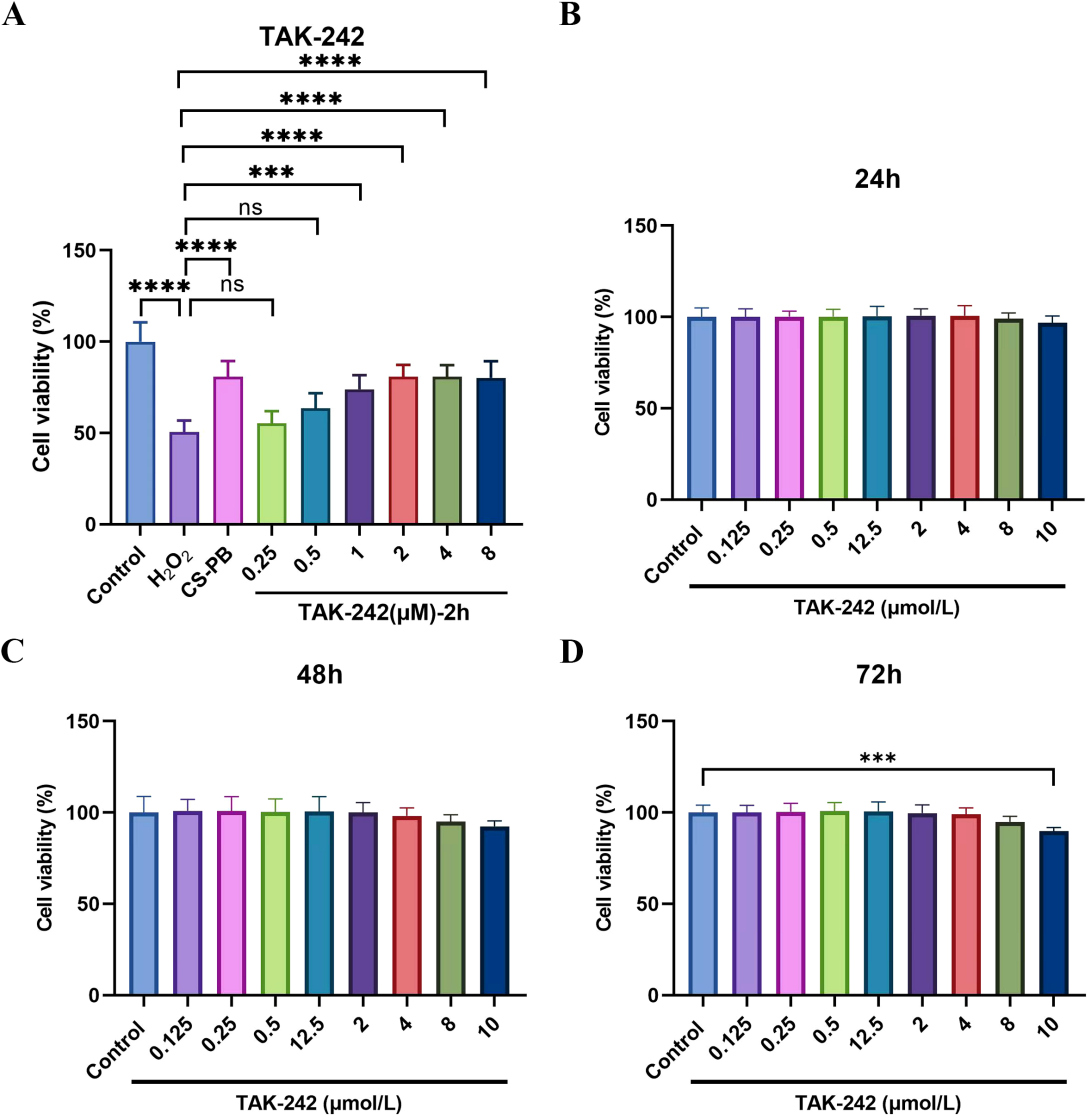
Cell viability of HEI-OC1 cells co-cultured with TAK-242 at different time. **(A)** Effects of different concentrations of TAK-242 pre-treatment for 2 h on HEI-OC1 cell viability injured by H_2_O_2_; **(B)** HEI-OC1 cell viability with TAK-242 co-culture at different concentrations for 24 h; **(C)** HEI-OC1 cell viability with TAK-242 co-culture at different concentrations for 48 h; **(D)** HEI-OC1 cell viability with TAK-242 co-culture at different concentrations for 72 h. ns indicates no significant difference between groups, *** indicates *P* < 0.001, and **** indicates *P* < 0.0001.

As previously indicated, preliminary experiments (data submitted for publication) revealed that escalating concentrations of H_2_O_2_ resulted in a significant dose-dependent decrease in HEI-OC1 cell viability, with an IC50 of approximately 200 μM. This concentration was subsequently employed to establish the oxidative stress model for further mechanistic investigations. CS-PB pretreatment with at 35 μg/mL for 4 h significantly mitigated H_2_O_2_-induced cell damage (**Figure 1A**, *P* < 0.0001), without, itself, showing a significant toxic effect on HEI-OC1 cells, on co-culture for up to 72 h.

#### 3.3.2 TAK-242 pretreatment reduced H_2_O_2_-induced cytotoxicity

TAK-242 pretreatment, 1-8 μM, significantly enhanced cell viability of the H_2_O_2_-injured cells, (*P* < 0.001-0.0001, **Figure 1A**), with the 2μM concentration showing a relatively favorable balance compared to lower concentrations; 0.25 and 0.5 μM which showed no significant protection. Higher concentrations; (4 and 8 μM) also increased cell viability, but with only a minor incremental benefit effect. Therefore, 2 μM was selected for further studies, as it reduced H_2_O_2_-induced cytotoxicity and optimized cell viability within the tested concentration range.

#### 3.3.3 Bio-safety of TAK-242

HEI-OC1 cells underwent co-culture with TAK-242 at various concentrations (0.125, 0.25, 0.5, 1, 2, 4, 8, and 10 μmol/L) for periods of 24, 48, and 72 hours. According to the CCK-8 assay results, cell viability did not significantly differ in most experimental groups from the control group at the 24- and 48-hour marks. A substantial drop in cell viability was observed with prolonged exposure (72 hours) to 10 μM TAK-242 compared to controls, showing cytotoxic effects that are dependent on both concentration and time (P < 0.001, **Figures 1B–D**). In contrast, our selected experimental conditions (2 μM TAK-242 for 2 h) showed no significant impact on cell viability, as supported by prior studies.

#### 3.3.4 Effect of TAK-242 and CS-PB on H_2_O_2_-injured HEI-OC1 cells

**Figure 2:**
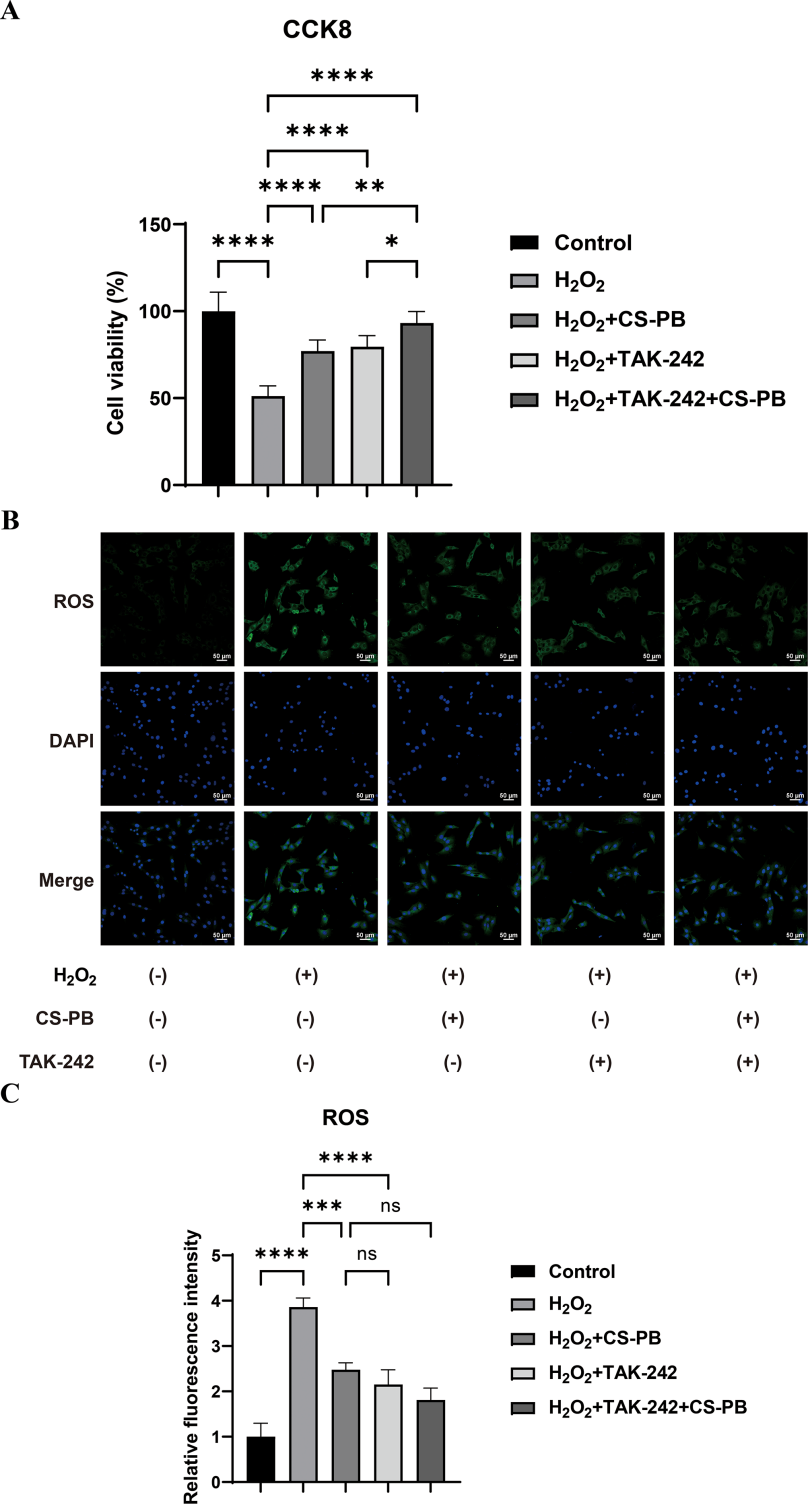
Effects of different treatments on HEI-OC1 cell survival rate and ROS levels. **(A)** Bar chart demonstrating the effect of different treatments on HEI-OC1 cell viability measured by CCK-8; **(B)** Intracellular ROS levels in HEI-OC1 cells determined by flow cytometry. Representative fluorescence images of ROS (green) and DAPI-stained nuclei (blue) and co-staining (merged); scale bar = 50 μm; **(C)** Quantitative analysis of relative ROS fluorescence intensity. Groups: Control (no H_2_O_2_/CS-PB/TAK-242), H_2_O_2_ (200 μM), H_2_O_2_ + CS-PB (35 μg/mL), H_2_O_2_ + TAK-242 (2 μM), H_2_O_2_ + TAK-242 + CS-PB. Data are presented as mean ± SD (n=3 biological replicates). ns = no significant difference; *P < 0.05; **P < 0.01; ***P < 0.001; ****P < 0.0001 (one-way ANOVA with Tukey’s *post-hoc* test). CCK-8, Cell Counting Kit-8; CS-PB, chitosan-Prussian blue nanozyme; ROS, reactive oxygen species; DAPI, 4’,6-diamidino-2-phenylindole.

H_2_O_2_ treatment markedly decreased cell viability relative to the control group, demonstrating induced cell damage. The H_2_O_2_ + CS-PB, H_2_O_2_ + TAK-242, and H_2_O_2_ + TAK-242 + CS-PB groups all exhibited significantly higher cell viability than the H_2_O_2_ group (*P* < 0.0001, **Figure 2A**). CS-PB and TAK-242 both significantly protected against H_2_O_2_-induced damage. Furthermore, The H_2_O_2_ + TAK-242 + CS-PB group showed a relatively high cell viability, which may reflect a modest enhancement in overall cell viability, although no statistically significant additive effects were observed in ROS levels, apoptosis, or oxidative stress markers.

#### 3.3.5 CS-PB reduced ROS levels in H_2_O_2_-injured HEI-OC1 cells

According to flow cytometry analysis using DCFH-DA staining (**Figure 2B**), there was a notable rise in intracellular ROS levels in HEI-OC1 cells damaged by H_2_O_2_ compared to the Control group (P < 0.0001; **Figure 2C**). Pretreatment with CS-PB (35 μg/mL for 4 h) or TAK-242 (2 μM for 2 h) similarly and significantly reduced ROS levels relative to the H_2_O_2_ group (both *P* < 0.001). No significant difference in ROS reduction was observed between the H_2_O_2_ + CS-PB, the H_2_O_2_ + TAK-242, and the H_2_O_2_ + TAK-242 + CS-PB groups (ns, *P* > 0.05). Based on relative fluorescence intensity quantification, CS-PB reduced H_2_O_2_-induced ROS elevation, from 3.8 to 2.4 by ~37% (**Figure 2C**).

#### 3.3.6 CS-PB reduced cell apoptosis, and oxidative damage in H_2_O_2_-injured HEI-OC1 cells

**Figure 3:**
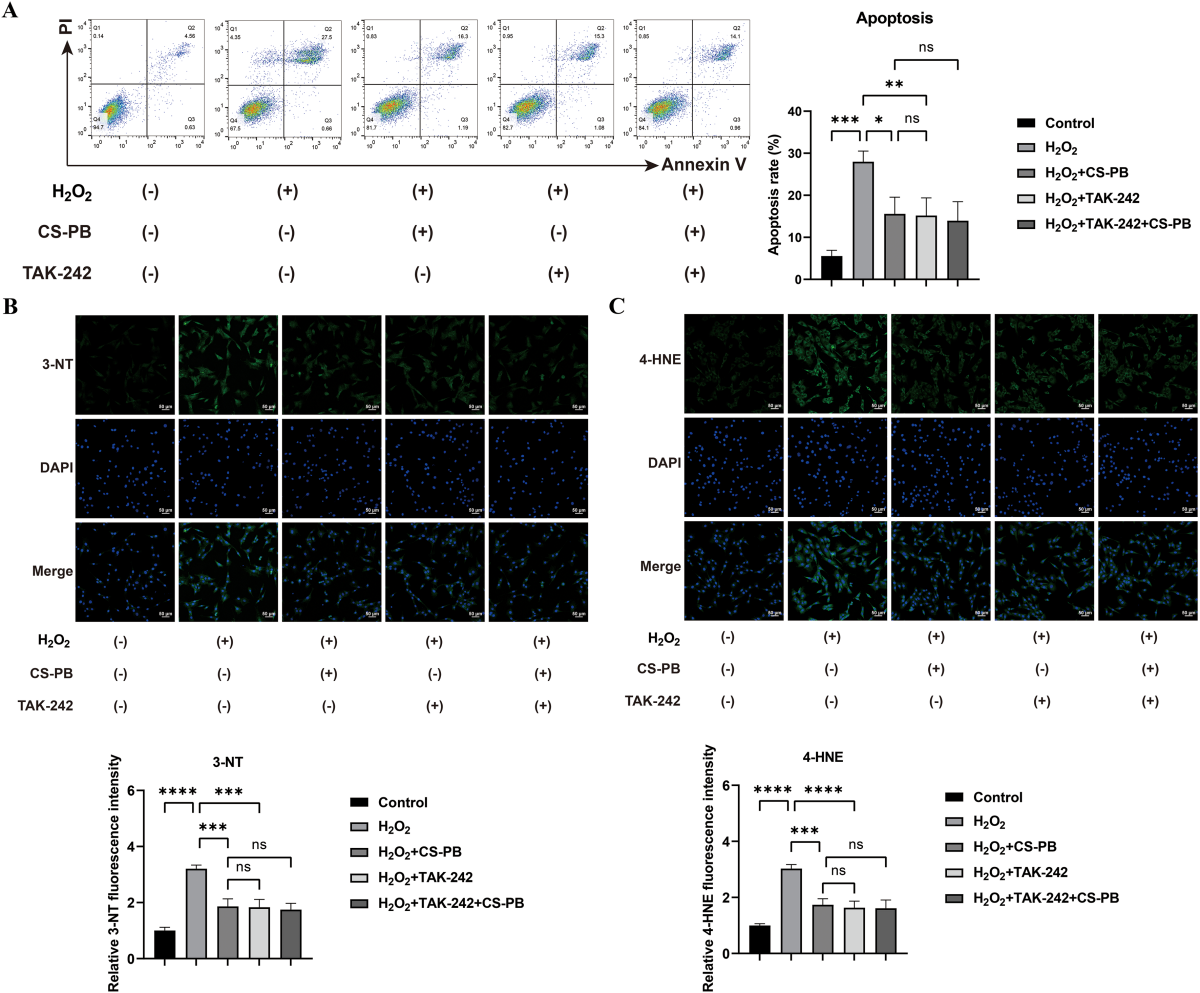
Flow cytometry analysis of cell apoptosis **(A)** and oxidative damage markers 3-NT **(B)** and 4-HNE **(C)** in HEI-OC1 cells of different groups. ns indicates no significant difference between groups, * indicates *P* < 0.05, ** indicates *P* < 0.01, *** indicates *P* < 0.001 and **** indicates *P* < 0.0001. 4-HNE, 4-hydroxynon-2-enal; 3-NT, 3-nitrotyrosine.

The flow cytometry data indicated a significant rise in cell apoptosis in the H_2_O_2_-injured group when compared with the blank control group (**Figure 3A**, P < 0.001). Apoptosis was significantly decreased (**Figure 3A**, *P* < 0.01–0-001) in the H_2_O_2_ + CS-PB and the H_2_O_2_ + TAK-242 and the H_2_O_2_ + TAK-242 + CS-PB groups compared to the H_2_O_2_ group. In the H_2_O_2_ group, there was a significant increase in the oxidative damage markers, 3-NT and 4-HNE (**Figures 3B, C**; P < 0.0001). In comparison to the H_2_O_2_ group, the fluorescence intensity of 3-nitrotyrosine (3-NT) was reduced by 41.96% in the H_2_O_2_+CS-PB group and by 42.83% in the H_2_O_2_+TAK-242 group. Similarly, the fluorescence intensity of 4-hydroxynonenal (4-HNE) decreased by 42.63% and 46.02%, respectively, with all differences being statistically significant (p < 0.001). No significant differences were observed in the levels of 3-NT and 4-HNE between the H_2_O_2_+CS-PB and H_2_O_2_+TAK-242 groups.

#### 3.3.7 CS-PB inhibited the TLR4/NF-κB pathway in HEI-OC1 cells

**Figure 4:**
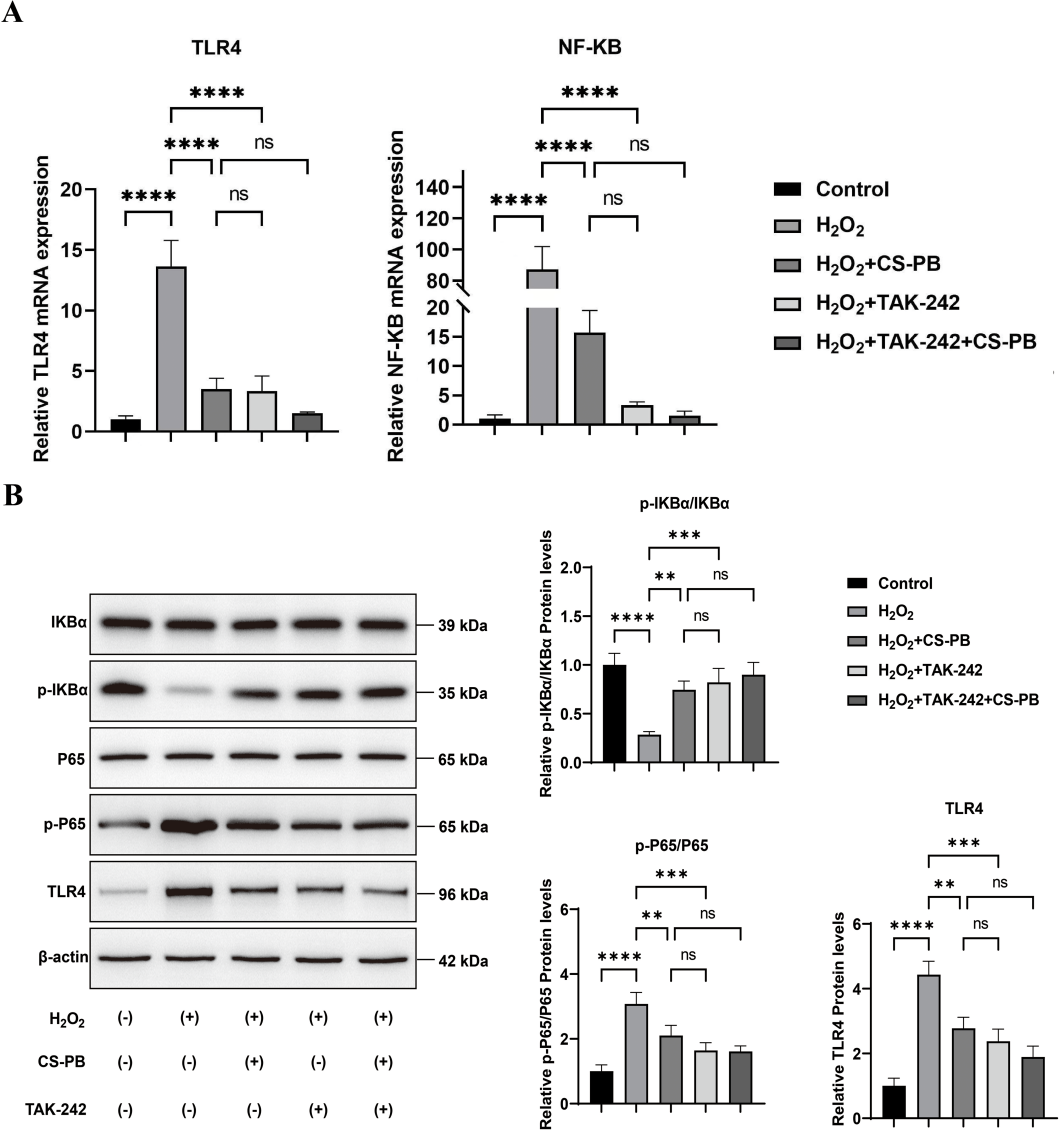
CS-PB inhibited the TLR4/NF-κB pathway in HEI-OC1 cells. **(A)** TLR4 and NF-κB pathway-related proteins in HEI-OC1 cells of different groups. (Y-axis is scaled to emphasize relative differences; all values are normalized to control group mean ± SD); **(B)** Western blot detection and quantitative analysis of IκBα, p-IκBα, P65, p-P65, and TLR4 proteins, with β-actin as a loading control. ns indicates no significant difference between groups, ** indicates *P* < 0.01, *** indicates *P* < 0.001, and **** indicates *P* < 0.0001. CS-PB, chitosan-Prussian blue nanozyme.

The qPCR results showed that CS-PB, similar to TAK-242, greatly reduced the expression of TLR4 and NF-κB proteins in HEI-OC1 cells damaged by H_2_O_2_ (**Figure 4A**, both P < 0.0001).The Western blotting results also showed that CS-PB (and TAK-242) notably reduced the expression of p-P65/P65 and TLR4 proteins (**Figure 4B**, both P < 0.01) while significantly increasing p-IκBα/IκBα protein levels (P < 0.01). The effects of combining CS-PB and TAK-242 were slightly greater but not significant. The findings suggest that CS-PB blocked the activation of the TLR4/NF-κB pathway.

#### 3.3.8 CS-PB regulated apoptosis-related proteins and inflammatory cytokines downstream of the TLR4/NF-κB pathway

**Figure 5:**
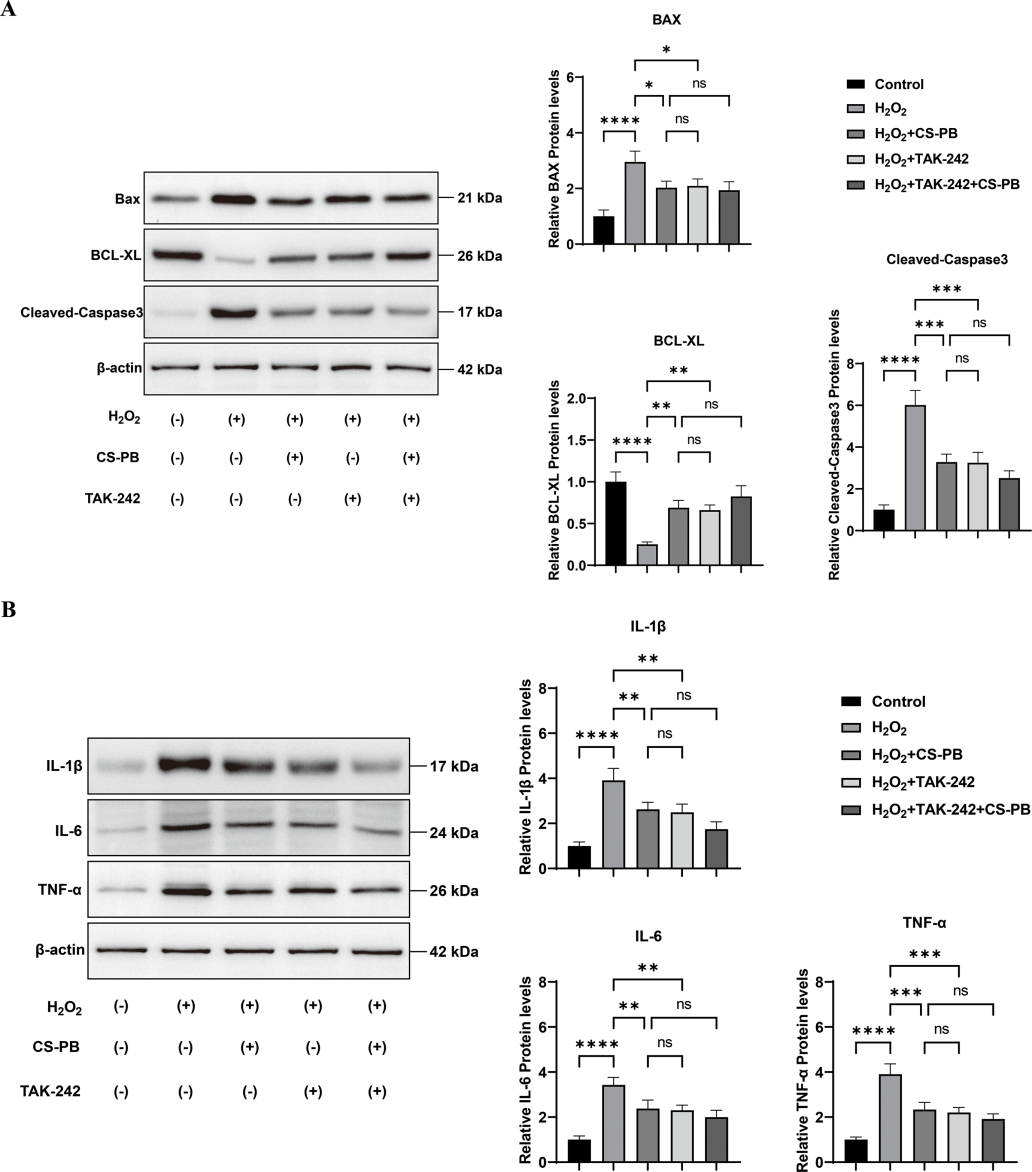
Western blot analysis of **(A)** apoptosis-related proteins (Bax, Bcl-XL, Cleaved-Caspase 3) and **(B)** inflammatory cytokines (IL-1β, IL-6, TNF-α) in HEI-OC1 cells of different groups. ns indicates no significant difference between groups, * indicates *P* < 0.05, ** indicates *P* < 0.01, *** indicates *P* < 0.001, and **** indicates *P* < 0.0001.

Western blot analysis demonstrated that CS-PB exerted a notable regulatory influence on downstream proteins in H_2_O_2_-damaged HEI-OC1 cells. Specifically, CS-PB significantly downregulated the expression of apoptosis-related proteins Bax and Cleaved-Caspase 3 (**Figure 5A**, P < 0.05 and P < 0.001, respectively) while upregulating Bcl-XL (P < 0.01). These findings suggest that CS-PB mitigates cellular apoptosis by inhibiting pro-apoptotic proteins and enhancing anti-apoptotic signaling pathways. Additionally, CS-PB markedly suppressed the expression of pro-inflammatory cytokines IL-1β, IL-6, and TNF-α (**Figure 5B**, P < 0.01 to P < 0.001). In line with previous findings, TAK-242 and the combination of CS-PB and TAK-242 and had no significantly different effects. Taken together, CS-PB exerted a downstream anti-inflammatory effect via the TLR4/NF-κB pathway.

### 3.4 CS-PB (2 mg/mL, selected concentration based on pilot screening) alleviated noise-induced hearing loss

#### 3.4.1 Development of animal model and pilot experiments for selection of the optimal CS-PB concentration

**Figure 6:**
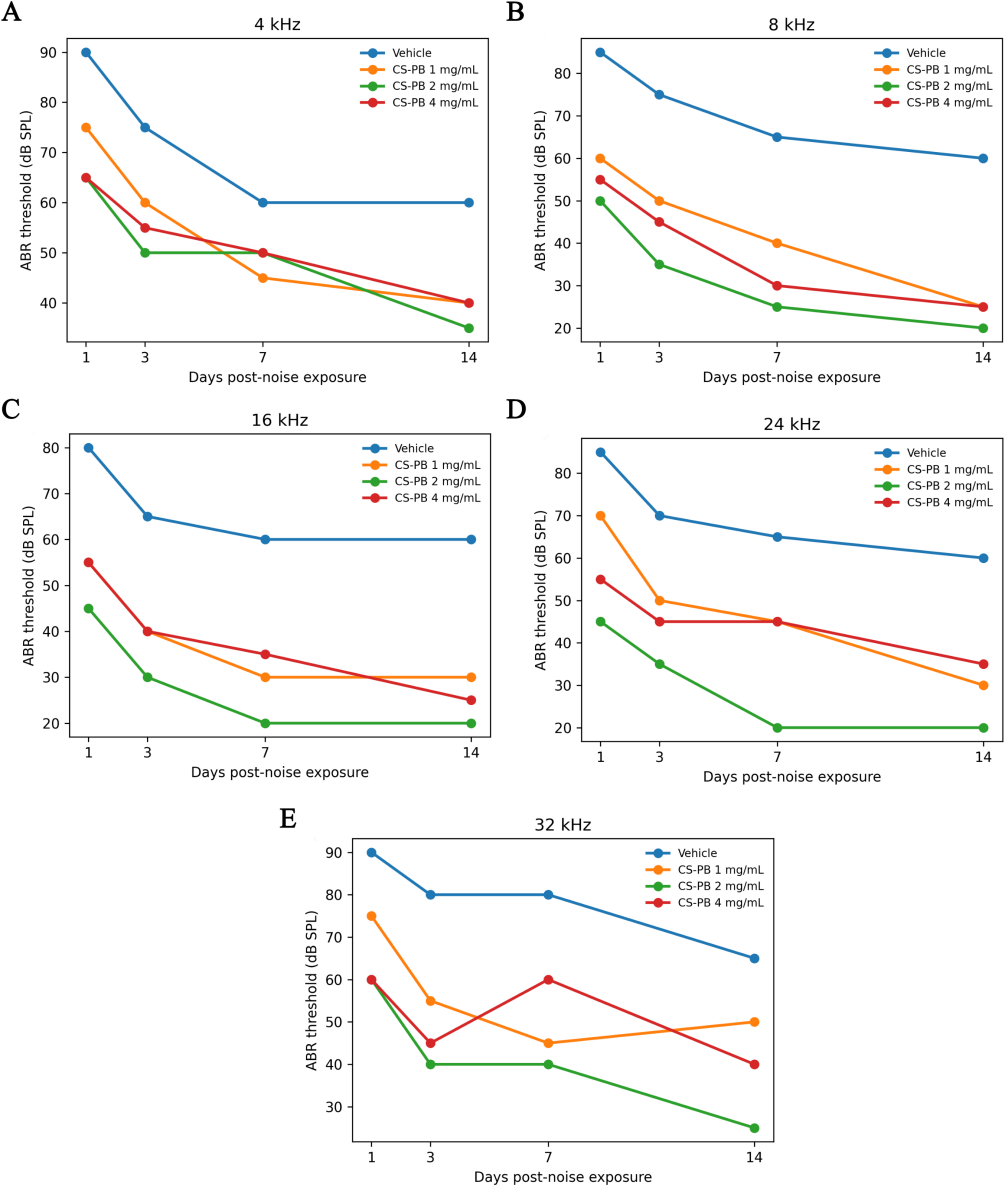
ABR thresholds (dB SPL) at different frequencies over time (Days 1, 3, 7, 14 post-noise exposure) across experimental groups. **(A)** ABR thresholds (dB SPL) at 4 kHz across experimental groups **(B)** ABR thresholds (dB SPL) at 8 kHz across experimental groups; **(C)** ABR thresholds (dB SPL) at 16 kHz across experimental groups; **(D)** ABR thresholds (dB SPL) at 24 kHz across experimental groups; **(E)** ABR thresholds (dB SPL) at 32 kHz across experimental groups. Vehicle control (blue), CS-PB 1 mg/mL (red), CS-PB 2 mg/mL (orange), and CS-PB 4 mg/mL (green). Shaded areas represent the standard error of the mean (SEM), and lines depict group mean thresholds. ABR, auditory brainstem response; CS-PB, chitosan-Prussian blue nanozyme.

In this study, a rat model for noise-induced hearing loss (NIHL) was developed, and the impact of locally applied CS-PB on hearing ability, activation of the cochlear TLR4/NF-κB pathway, and expression of inflammatory cytokines was assessed at 6, 24, and 72 hours after noise exposure. A significant main effect of Group was detected across all tested frequencies (4–32 kHz; *P* = 0.048 to 0.00088), indicating dose-dependent variation in ABR thresholds. The main effect of Time was also significant at every frequency (4 kHz *P* = 0.028; 8 kHz *P* = 0.007; 16 kHz *P* = 0.0499; 24 kHz *P* = 0.039; 32 kHz P = 0.0028), showing an overall recovery trend over days 1-14 (**Figure 6**). Although there was no statistically significant interaction between Group and Time (P > 0.79), the temporal trajectories of different CS-PB doses diverged visibly, suggesting biologically meaningful separation (see line plots and pairwise comparisons **Table 1**, **Figure 6**).

**Table 1:** LMM results for ABR thresholds at 4–32 kHz, assessing effects of group (vehicle control, CS-PB 1, 2, and 4 mg/mL), time (1, 3, 7 and 14 days post-noise), and their interaction.

Frequency (kHz)	Effect	F-value	*P*-value
4	Group	5.83	0.0069
4	Time	4.50	0.0281
4	Group × Time	0.23	0.9853
8	Group	3.29	0.0477
8	Time	5.83	0.0069
8	Group × Time	0.12	0.9985
16	Group	9.24	0.0009
16	Time	4.50	0.0499
16	Group × Time	0.26	0.9691
24	Group	3.44	0.0421
24	Time	4.00	0.0390
24	Group × Time	0.36	0.9371
32	Group	4.50	0.0180
32	Time	12.50	0.0027
32	Group × Time	0.56	0.7923

“Ear” was a random intercept. *P* < 0.05 denoted significance. LMM, Linear mixed-effects model; ABR, auditory brainstem response; CS-PB, chitosan-Prussian blue nanozyme.

##### 3.4.1.1 Protective effect of CS-PB 2 mg/mL (target dose) at day 14

At 14 days post- -noise exposure (the final observation time point), comparison between the 2 mg/mL CS-PB and the vehicle control groups revealed consistent ABR threshold reductions across frequencies (**Supplementary Figure 18**; **Table 2**; **Supplementary Table 1**). Relatively consistent reductions in ABR thresholds (approximately 40 dB on average) were observed at 8–24 kHz, with individual variability observed at the ear level. In contrast, the 1 mg/mL CS-PB group only exhibited a threshold reduction of around 30 dB at 16 kHz and 24 kHz, with unstable reductions at other frequencies. The 4 mg/mL CS-PB group showed threshold reductions of 25–35 dB across all frequencies, with a smaller protective effect than the 2 mg/mL group. Taken together, 2 mg/mL CS-PB produced the most pronounced and uniform protective effect across mid-to-high frequencies, supporting its selection as the working concentration for subsequent experiments.

**Table 2:** ABR threshold shifts at Day 14 post-noise exposure in the CS-PB pilot study.

Frequency (kHz)	Vehicle control (dB) mean ± SD	CS-PB 1 mg/mL (dB) mean ± SD	CS-PB 2 mg/mL (dB) mean ± SD	CS-PB 4 mg/mL (dB) mean ± SD
4 kHz	60 ± 14.1	40 ± 0	35 ± 7.1	40 ± 14.1
8 kHz	60 ± 0	25 ± 7.1	20 ± 0	25 ± 7.1
16 kHz	60 ± 0	30 ± 0	20 ± 0	25 ± 7.1
24 kHz	60 ± 0	30 ± 0	20 ± 0	35 ± 21.2
32 kHz	60 ± 7.1	50 ± 14.1	25 ± 7.1	40 ± 28.3

Data are presented as mean ± SD (n = 2 ears per group).

Because this pilot experiment included only two ears per group, identical values between ears result in zero standard deviation for some frequencies.

This pilot study was exploratory, intended to identify a working concentration for subsequent experiments rather than for formal statistical inference.

#### 3.4.2 CS-PB suppressed noise-induced activation of the cochlear TLR4/NF-κB pathway

**Figure 7:**
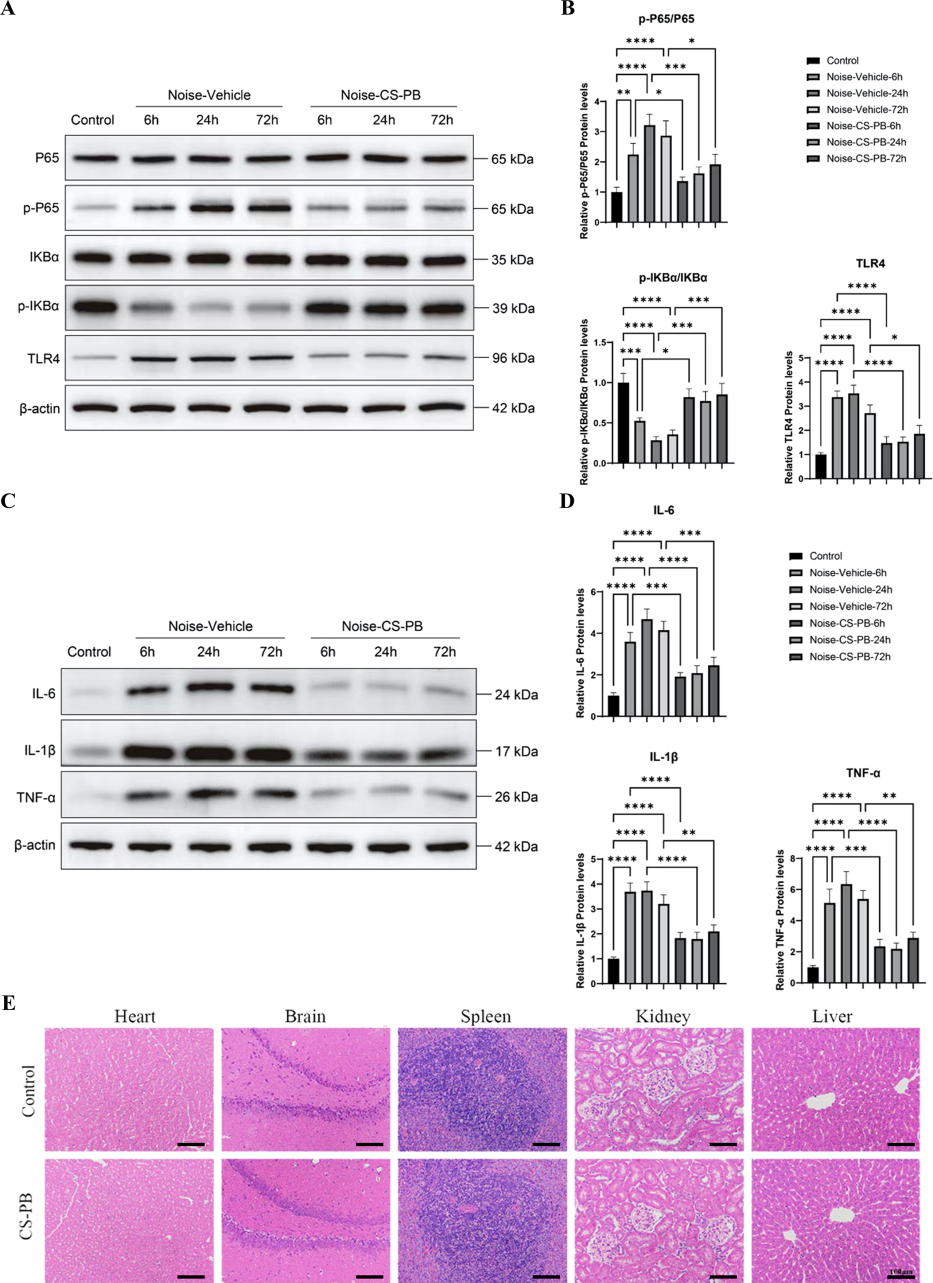
Effects of noise exposure on TLR4/NF-κB signaling pathway activation and inflammatory cytokine expression in the cochlea, and biosafety assessment of CS-PB. **(A)** Representative Western blot images of TLR4, IκBα, p-IκBα, P65, p-P65, and β-actin (loading control) in cochlear tissues at 6, 24, and 72 h post-noise exposure; **(B)** Quantitative analysis of relative protein levels (mean ± SEM, normalized to loading control); **(C)** Representative Western blot images of IL-1β, IL-6, TNF-α, and β-actin (loading control) in cochlear tissues at 6, 24, and 72 h post-noise exposure; **(D)** Quantitative analysis of relative cytokine levels (mean ± SEM, normalized to loading control); **(E)** Representative images of H&E stained heart, brain, spleen, kidney and liver sections in CS-PB and saline Control treated rats. Statistical comparisons: * indicates *P* < 0.05, ** indicates *P* < 0.01, *** indicates *P* < 0.001, and **** indicates *P* < 0.0001 vs. Noise-Vehicle. CS-PB, chitosan-Prussian blue nanozyme.

We investigated the impact of noise exposure on the TLR4/NF-κB signaling pathway by analyzing cochlear p-P65/P65, p-IκBα/IκBα, and TLR4 protein levels at 6, 24, and 72 hours post-exposure (**Figures 7A, B**). In the Noise-Vehicle group, noise exposure induced a significant time-dependent stimulation of pathway activation: increased p-P65/P65 and decreased p-IκBα/IκBα. P-P65/P65 levels rose by 2.2-fold at 6 h (all *P* < 0.01 vs. Control), peaked at 24 h with 3.2-fold increases (all *P* < 0.0001), and remained elevated at 72 h (2.9-fold increases, all *P* < 0.001). Similarly, TLR4 expression increased significantly 3.4-fold at 6 h (*P* < 0.001 vs. Control), peaked at 24 h with a 3.5-fold increase (*P* < 0.0001), and persisted at high levels at 72 h (1.9-fold increase, *P* < 0.001). CS-PB pretreatment markedly attenuated these changes. At 24 h post-noise, CS-PB significantly reduced p-P65/P65 by 59% (from 3.2-fold to 1.3-fold of Control), increased p-IκBα/IκBα by 58% (from 0.3-fold to 0.47-fold of Control), and decreased TLR4 by 51% (from 3.5-fold to 1.7-fold of Control) (all *P* < 0.001 vs. Noise-Vehicle). By 72 h, p-P65/P65 (1.4-fold of Control) and p-IκBα/IκBα (0.9-fold of Control) levels were restored to near-control values (*P* > 0.05 vs. Control), while TLR4 expression (1.9-fold of Control) remained 42% lower than in the Noise-Vehicle group (*P* < 0.001). Notably, the degree of CS-PB-mediated suppression of pathway activation correlated strongly with the reduction of inflammatory cytokine levels (r = 0.89 for p-P65 vs. IL-1β, r = 0.87 for p-IκBα vs. IL-6, r = 0.88 for TLR4 vs. TNF-α; all *P* < 0.001), confirming that CS-PB exerted cochlear anti-inflammatory effects through inhibition of the TLR4/NF-κB pathway.

#### 3.4.3 CS-PB reduced noise-induced inflammatory cytokine expression in the cochlea

The Western blot analysis results for cochlear IL-1β, IL-6, and TNF-α levels, significant in NIHL pathogenesis, are shown in **Figures 7C, D**. In the Noise-Vehicle group, exposure to noise resulted in a significant time-dependent increase in the levels of various cytokines. Specifically, the levels of interleukin-1 beta (IL-1β) protein demonstrated a 3.7-fold increase at 6 hours (P < 0.001 compared to the control group), reached a peak of 3.7-fold upregulation at 24 hours (P < 0.0001 compared to the control group), and remained significantly elevated at 72 hours with a 3.2-fold increase (P < 0.001 compared to the control group).In a similar manner, interleukin-6 (IL-6) concentrations exhibited a 3.6-fold elevation at the 6-hour mark (P < 0.001 relative to the Control group), attained a peak increase of 4.7-fold at 24 hours (P < 0.0001 relative to the Control group), and persisted at elevated levels with a 2.5-fold increase at 72 hours (P < 0.001 relative to the Control group).The levels of tumor necrosis factor-alpha (TNF-α) exhibited a 5.1-fold elevation at 6 hours (P < 0.001 relative to the control group), reached a peak with a 6.3-fold upregulation at 24 hours (P < 0.0001 relative to the control group), and persisted at elevated levels with a 2.9-fold increase at 72 hours (P < 0.001 relative to the control group).The pretreatment with CS-PB markedly mitigated the upregulation of cytokines. At 24 hours post-noise exposure, there was a reduction in IL-1β levels by 54% (from a 3.7-fold to a 1.7-fold increase relative to the Control), IL-6 levels by 59% (from a 4.7-fold to a 1.9-fold increase relative to the Control), and TNF-α levels by 67% (from a 6.3-fold to a 2.1-fold increase relative to the Control), with all reductions being statistically significant (P < 0.001 compared to the Noise-Vehicle group).At the 72-hour mark, IL-6 (1.8-fold of Control) and TNF-α (2.2-fold of Control) levels had returned to values similar to the Control group (P > 0.05), but IL-1β levels (2.1-fold of Control) were 34% less than those in the Noise-Vehicle group. Importantly, the extent of cytokine suppression by CS-PB was strongly correlated with decreased p-P65/P65 levels (r = 0.89 for IL-1β, r = 0.87 for IL-6, r = 0.88 for TNF-α; all P < 0.001), substantiating that its anti-inflammatory effects in the cochlea are mediated through the inhibition of the TLR4/NF-κB signaling pathway.

#### 3.4.4 Biosafety assessment of CS-PB

The histopathological examination showed no notable pathological changes in the heart, brain, spleen, kidneys, or liver of rats following local cochlear administration of CS-PB (**Figure 7E**). These findings indicate that CS-PB is biosafe when locally applied to the cochlea. No significant variations in ALT, AST, BUN, Cr, and CK levels were observed between the control and CS-PB groups according to the serum biochemical analysis. (**Table 3**). These findings suggest that CS-PB administration exerts no detectable hepatic, renal or muscle toxicity.

**Table 3:** Serum levels of ALT, AST, BUN, Cr, and CK in the control and CS-PB groups.

Grouping	ALT (U/L)	AST (U/L)	BUN (mg/dl)	Cr (umol/L)	CK (U/L)
Control	26.1 ± 1.6	134.6 ± 28.4	17.9 ± 2.5	40.2 ± 2.1	915.6 ± 134.5
CS-PB	23.8 ± 0.7	131.7 ± 16.7	18.4 ± 2.2	38.4 ± 1.9	926.8 ± 155.4

Data are presented as mean ± SD (n = 10 per group). No significant differences were observed between groups (one-way ANOVA with Tukey’s *post-hoc* test). ALT, alanine aminotransferase; AST, aspartate aminotransferase; BUN, blood urea nitrogen; Cr, creatinine; CK, creatine kinase; CS-PB, chitosan-Prussian blue nanozyme.

## 4 Discussion

This study demonstrates that CS-PB effectively mitigated oxidative stress and inflammation in auditory cells through a dual mechanism: ROS scavenging and inhibition of the TLR4/NF-κB pathway. These mechanisms position CS-PB as a potential therapeutic approach for sensorineural hearing loss and other oxidative stress-related auditory disorders.

In pilot studies, 2 mg/mL CS-PB significantly reduced ABR thresholds in rats exposed to broadband noise, particularly at mid-to-high frequencies (8–24 kHz). This selected concentration was shown to reduce ROS by 37%, *in vitro*, demonstrating its antioxidative capacity while it was also shown to regulate apoptosis-related proteins such as Bax and Cleaved-caspase-3 in HEI-OC1 cochlear cells. These effects can be attributed to synergy between the SOD- and CAT-like activities of PBNP protecting cochlear cells from oxidative stress-induced injury (11), while chitosan provides a biocompatible matrix stabilizing nanoparticles but also contributing anti-inflammatory protection via intrinsic free-radical-scavenging and TLR4/NF-κB-modulation (1, 2, 10).

Our results align with prior evidence of the ROS-scavenging efficiency of PBAs with multi-enzyme mimetic functions that collectively suppress oxidative stress (11). The dual regulation of oxidative and inflammatory pathways suggests that CS-PB may disrupt self-amplifying ROS-inflammation feedback, providing a promising strategy for cochlear protection. Previous studies have also indicated that chitosan-based nanocarriers enhance dispersion, and cellular uptake of nanoparticles improving drug absorption and enabling controlled release (10), supporting the concept that chitosan acts as a functional enhancer as well as a stabilizer in nanozyme composites. We found that CS-PB suppressed apoptosis from 27.5% in the H_2_O_2_ group to 14.1% (*P* < 0.01) by upregulating the anti-apoptotic protein Bcl-xL and downregulating pro-apoptotic Bax. This effect is consistent with the reported role of chitosan in inhibition of apoptosis by regulating mitochondrial membrane potential (12, 13), together with the antioxidant effects of PBAs (11).

CS-PB reduced inflammatory cytokines (TNF-α, IL-1β, IL-6) by inhibiting the TLR4/NF-κB signaling pathway, which plays a crucial role in controlling the body’s inflammatory response to infections and injuries (14). Our study revealed that CS-PB effectively downregulated TLR4 expression and inhibited the phosphorylation of downstream molecules, specifically p-IκBα/IκBα and p-P65/P65. This inhibition represents a critical step in the activation of NF-κB, facilitating its nuclear translocation and the subsequent production of pro-inflammatory cytokines (5).

TAK-242, a well-characterized selective TLR4 antagonist, blocks TLR4 signaling by interfering with the interaction between TLR4 and its adaptor molecules. Consistent with previous reports, TAK-242 pretreatment significantly attenuated oxidative stress and inflammatory responses in H_2_O_2_-injured HEI-OC1 cells. Notably, CS-PB treatment produced effects comparable to those observed with TAK-242, suggesting that CS-PB may exert its anti-inflammatory activity largely through modulation of the same TLR4/NF-κB signaling pathway.

Interestingly, although the combination of CS-PB and TAK-242 resulted in a modest improvement in overall cell viability, no significant additional reductions were observed in ROS levels, apoptosis, or oxidative damage markers (3-NT and 4-HNE) compared with either treatment alone (**Figures 4**–**6**). This observation may be explained by the mechanistic overlap between CS-PB and TAK-242, as both agents target the same TLR4/NF-κB signaling cascade. Once this pathway is effectively inhibited by either agent, further suppression through combined treatment may reach a ceiling effect, limiting additional reductions in downstream oxidative and apoptotic markers.

These findings provide further evidence that the protective effects of CS-PB are closely associated with inhibition of the TLR4/NF-κB pathway. Moreover, the slight improvement in cell viability observed in the combination group may reflect complementary protective mechanisms of CS-PB, including its intrinsic ROS-scavenging capacity derived from the Prussian blue nanozyme component, in addition to its anti-inflammatory activity mediated by chitosan.

This study is the first to demonstrate the cross-regulation of TLR4/NF-κB and oxidative stress in auditory cells. In NIHL mice, TLR4 macrophage activation worsened cochlear pathology. The cochlea was previously considered to be immune-privileged but resident macrophages were shown to alter in shape, to multiply, and to move to hair cell areas, causing additional damage and loss of outer hair cells and ribbon synapses (18) significantly contributing to the immune response after acoustic trauma in mice. We showed that CS-PB was able to break the vicious cycle of oxidative stress-inflammation through dual inhibition of ROS and TLR4 signaling mirroring the findings of a recent interdisciplinary innovation (19), highlighting the potential of nanocarriers to modulate multiple pathways synergistically.

The biocompatibility of CS-PB is critically important for clinical translation. *In vitro*, CCK-8 assay showed no significant cytotoxicity after 72 h exposure to CS-PB 35 μg/mL. *In vivo* studies revealed no pathological changes in the main organs of rats, with normal liver and kidney biochemistry, supporting the low immunogenicity of chitosan as a food-grade polysaccharide (20, 21). *In vivo*, CS-PB (2 mg/mL), locally delivered to the cochlea, provided significant protection against noise-induced hearing loss in rats, highlighting its potential for future clinical applications e.g. in treating sensorineural hearing loss or other oxidative stress-related disorders.

The CS-PB nanoscale size (100-200 nm) facilitates efficient cellular uptake via endocytosis, making it suitable for local administration (e.g., intra-tympanic injection) to target inner ear hair cells. In a similar vein, poly (D, L-lactide-co-glycolide acid) (PLGA) nanoparticles have been investigated for their potential to enhance the local bioavailability of therapeutics administered to the inner ear. These nanoparticles demonstrate efficacy in traversing the round window membrane (RWM), thereby significantly enhancing drug distribution and perilymph concentrations. Consequently, PLGA nanoparticles represent a promising strategy for the treatment of inner ear conditions (22). Exploration of novel antioxidant delivery systems has gained significant attention in recent years, particularly in the context of enhancing the efficacy and bioavailability of traditional antioxidants such as N-acetylcysteine (NAC) (23, 24) which require frequent administration. In contrast, CS-PB offers prolonged activity and targeted delivery. In addition, the amino groups of chitosan potentially enable further functionalization with targeting ligands (e.g., hair cell-specific antibodies), enhancing delivery precision -an approach analogous to the lipid nanoparticle-mediated CRISPR delivery (25).

We acknowledge some limitations of our studies: we investigated a single cellular auditory cell model, involving H_2_O_2_ injury in depth. Additionally, this study concerns the combined nanozyme behavior and therapeutic potential of CS-PB, rather than the isolated effects of its individual constituents. A limited number of rats were studied, with no further *in vivo* validation of CS-PB in other hearing loss animal models (e.g., other species, other noise exposures or ototoxic, drug-induced injury). Larger, more comprehensive, studies are required. Future research should evaluate the pharmacokinetics, biodistribution, and long-term safety of CS-PB *in vivo*. Additionally, the potential toxicity of iron ion release from Prussian blue requires optimization through surface modification (e.g., PEGylation) (26). Despite these limitations, this work establishes a novel paradigm for combination nanoenzyme/anti-inflammatory/antioxidant therapy, providing a mechanistic foundation for developing multifunctional nanodrugs against neurodegenerative and inflammatory diseases.

## 5 Conclusions

**Figure 8:**
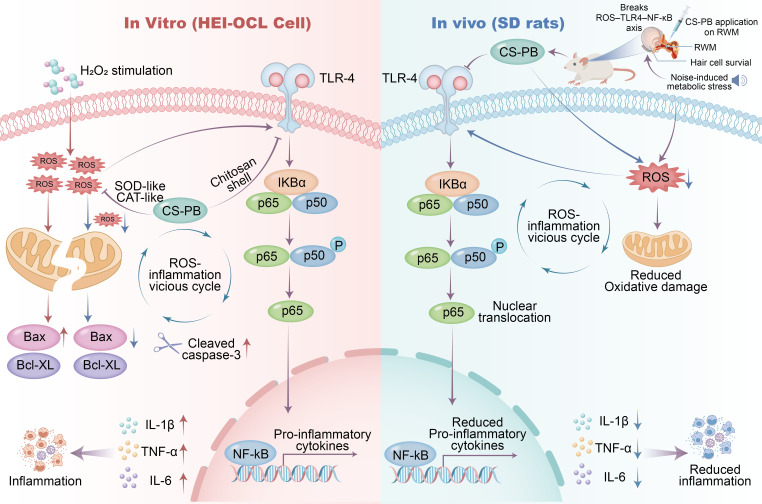
Schematic diagram of the mechanism of action of CS-PB *in vivo* and *in vitro*.

Both *in vitro* and *in vivo* studies showed (see **Figure 8** for schematic representation of the mechanism of action) that CS-PB offers considerable protection to cochlear cells, resulting in better auditory thresholds in rats experiencing noise-induced hearing loss. CS-PB represents a promising non-toxic, nanotherapeutic strategy that combines ROS scavenging with TLR4/NF-κB pathway inhibition to protect auditory cells from oxidative stress and inflammation. The dual-mechanism design and safety results boost the potential of CS-PB as a new treatment method for sensorineural hearing loss and other oxidative stress-related conditions. By integrating natural polysaccharides with artificial nanozyme activity, this study expands the application of nanozymes in sensory organ protection, showcasing the versatility of nanozyme-based therapies.

## Data Availability

The original contributions presented in the study are included in the article/**Supplementary Material**. Further inquiries can be directed to the corresponding author.

## References

[1] Rajkumar M Govindaraj P Vimala K Thangaraj R Kannan S . Chitosan/PLA-loaded magnesium oxide nanocomposite to attenuate oxidative stress, neuroinflammation and neurotoxicity in rat models of Alzheimer’s disease. Metab Brain Dis. (2024) 39:487–508. doi: 10.1007/s11011-023-01336-x. PMID: 38085467

[2] Ngo DN . Chitin, chitosan, and their derivatives against oxidative stress and inflammation, and some applications. In: Kim SK , editor.Seafood processing by-products: trends and applications. Springer, New York, NY (2014). p. 389–405. doi: 10.1007/978-1-4614-9590-1_19

[3] Park C Thein P Kalinec G Kalinec F . HEI-OC1 cells as a model for investigating prestin function. Hear Res. (2016) 335:9–17. doi: 10.1016/j.heares.2016.02.001. PMID: 26854618

[4] Takashima K Matsunaga N Yoshimatsu M Hazeki K Kaisho T Uekata M . Analysis of binding site for the novel small-molecule TLR4 signal transduction inhibitor TAK-242 and its therapeutic effect on mouse sepsis model. Br J Pharmacol. (2009) 157:1250–62. doi: 10.1111/j.1476-5381.2009.00297.x. PMID: 19563534 PMC2743844

[5] Christian F Smith EL Carmody RJ . The regulation of NF-κB subunits by phosphorylation. Cells. (2016) 5:12. doi: 10.3390/cells5010012. PMID: 26999213 PMC4810097

[6] Matsunaga N Tsuchimori N Matsumoto T Ii M . TAK-242 (resatorvid), a small-molecule inhibitor of Toll-like receptor (TLR) 4 signaling, binds selectively to TLR4 and interferes with interactions between TLR4 and its adaptor molecules. Mol Pharmacol. (2011) 79:34–41. doi: 10.1124/mol.110.068064. PMID: 20881006

[7] Zhuang C Ni S Yang ZC Liu RP . Oxidative stress induces chondrocyte apoptosis through caspase-dependent and caspase-independent mitochondrial pathways and the antioxidant mechanism of Angelica sinensis polysaccharide. Oxid Med Cell Longevity. (2020) 2020:3240820. doi: 10.1155/2020/3240820. PMID: 33224431 PMC7669361

[8] Song Q Zhou ZJ Cai S Chen Y Chen P . Oxidative stress links the tumour suppressor p53 with cell apoptosis induced by cigarette smoke. Int J Environ Health Res. (2022) 32:1745–55. doi: 10.1080/09603123.2021.1910211. PMID: 33825597

[9] Nkpaa KW Awogbindin IO Amadi BA Abolaji AO Adedara IA Wegwu MO . Ethanol exacerbates manganese-induced neurobehavioral deficits, striatal oxidative stress, and apoptosis via regulation of p53, caspase-3, and Bax/Bcl-2 ratio-dependent pathway. Biol Trace Elem Res. (2019) 191:135–48. doi: 10.1007/s12011-018-1587-4. PMID: 30488170

[10] Buttke TM Sandstrom PA . Oxidative stress as a mediator of apoptosis. Immunol Today. (1994) 15:7–10. doi: 10.1016/0167-5699(94)90018-3. PMID: 8136014

[11] Zhou R Wang P Guo Y Dai X Xiao S Fang Z . PRussian blue analogue nanoenzymes mitigate oxidative stress and boost bio-fermentation. Nanoscale. (2019) 11:19497–505. doi: 10.1039/C9NR04951G. PMID: 31553036

[12] Wang C Ren G Yuan B Zhang W Lu M Liu J . Enhancing enzyme-like activities of pRussian blue analog nanocages by molybdenum doping: Toward cytoprotecting and online optical hydrogen sulfide monitoring. Anal Chem. (2020) 92:7822–30. doi: 10.1021/acs.analchem.0c01028. PMID: 32378404

[13] Li J He J Yu C . Chitosan oligosaccharide inhibits LPS-induced apoptosis of vascular endothelial cells through the BKCa channel and the p38 signaling pathway. Int J Mol Med. (2012) 30:157–64. doi: 10.3892/ijmm.2012.954. PMID: 22469656

[14] Zhang C Yu L Zhou Y Zhao Q Liu SQ . Chitosan oligosaccharides inhibit IL-1β-induced chondrocyte apoptosis via the P38 MAPK signaling pathway. Glycoconjugate J. (2016) 33:735–44. doi: 10.1007/s10719-016-9667-1. PMID: 27178341

[15] Ye S Zheng Q Zhou Y Bai B Yang D Zhao Z . Chlojaponilactone B attenuates lipopolysaccharide-induced inflammatory responses by suppressing TLR4-mediated ROS generation and NF-κB signaling pathway. Molec (Basel Switzerl). (2019) 24:3731. doi: 10.3390/molecules24203731. PMID: 31623197 PMC6832138

[16] Gargiulo S Gamba P Testa G Rossin D Biasi F Poli G . Relation between TLR4/NF-κB signaling pathway activation by 27-hydroxycholesterol and 4-hydroxynonenal, and atherosclerotic plaque instability. Aging Cell. (2015) 14:569–81. doi: 10.1111/acel.12322. PMID: 25757594 PMC4531071

[17] Zhang Y Li Q Han C Geng F Zhang S Qu Y . Superoxide dismutase@zeolite imidazolate framework-8 attenuates noise-induced hearing loss in rats. Front Pharmacol. (2022) 13:885113. doi: 10.3389/fphar.2022.885113. PMID: 35662706 PMC9159373

[18] Pan J Wang K Qu J Chen D Chen A You Y . Activated tissue-resident macrophages contribute to hair cell insults in noise-induced hearing loss in mice. Commun Biol. (2024) 7:1078. doi: 10.1038/s42003-024-06768-4. PMID: 39223249 PMC11368919

[19] Shou X Chen C Ying H Liu Z Zeng L Li Q . Biomimetic MOF nanocarrier-mediated synergistic delivery of mitochondria and anti-inflammatory miRNA to alleviate acute lung injury. Adv Sci (Weinheim Baden-Wurttemberg Germany). (2025) 12:e2416594. doi: 10.1002/advs.202416594. PMID: 39999302 PMC12021094

[20] Bonnaud M Weiss J McClements DJ . Interaction of a food-grade cationic surfactant (lauric arginate) with food-grade biopolymers (pectin, carrageenan, xanthan, alginate, dextran, and chitosan). J Agric Food Chem. (2010) 58:9770–7. doi: 10.1021/jf101309h. PMID: 20684547

[21] Ahmed R Wang M Qi Z Hira NU Jiang J Zhang H . Pickering emulsions based on the pH-responsive assembly of food-grade chitosan. ACS Omega. (2021) 6:17915–22. doi: 10.1021/acsomega.1c01490. PMID: 34308026 PMC8295998

[22] Cai H Wen X Wen L Tirelli N Zhang X Zhang Y . Enhanced local bioavailability of single or compound drugs delivery to the inner ear through application of PLGA nanoparticles via round window administration. Int J Nanomed. (2014) 9:5591–601. doi: 10.2147/IJN.S72555. PMID: 25489245 PMC4257110

[23] Puri V Chaudhary KR Singh A Singh C . Inhalation potential of N-acetylcysteine loaded PLGA nanoparticles for the management of tuberculosis: *In vitro* lung deposition and efficacy studies. Curr Res Pharmacol Drug Discov. (2022) 3:100084. doi: 10.1016/j.crphar.2022.100084. PMID: 35112077 PMC8790477

[24] Zhang S Asghar S Yu F Hu Z Ping Q Chen Z . The enhancement of N-acetylcysteine on intestinal absorption and oral bioavailability of hydrophobic curcumin. Eur J Pharm Sci. (2020) 154:105506. doi: 10.1016/j.ejps.2020.105506. PMID: 32763460

[25] Xiao Z Li Y Xiong L Liao J Gao Y Luo Y . Recent advances in anti-atherosclerosis and potential therapeutic targets for nanomaterial-derived drug formulations. Adv Sci (Weinheim Baden-Wurttemberg Germany). (2023) 10:e2302918. doi: 10.1002/advs.202302918. PMID: 37698552 PMC10582432

[26] Cheng L Gong H Zhu W Liu J Wang X Liu G . PEGylated pRussian blue nanocubes as a theranostic agent for simultaneous cancer imaging and photothermal therapy. Biomaterials. (2014) 35:9844–52. doi: 10.1016/j.biomaterials.2014.09.004. PMID: 25239041

