# Supplementary material for: Studies of chitosan-Prussian blue nanozyme in auditory protection: from cellular mechanisms to in vivo validation
Source: Front Immunol. 2026 Apr 21;17:1758392. doi: 10.3389/fimmu.2026.1758392 (PMC13139334; doi:10.3389/fimmu.2026.1758392)
Supplement: Supplementary file 1 [file Table1.doc]

**Supplementary Materials**

**Supplementary Method**

**Physicochemical characterization**

**Morphology and size analysis (TEM/SEM and ImageJ statistics)**

CS-PB powder was dispersed in ultrapure water, sonicated for 10 min, and dropped onto copper grids (TEM) or silicon wafers (SEM). After natural drying, samples were characterized by TEM/SEM. Particle sizes were quantified from TEM images using ImageJ by measuring at least 100 nanoparticles, and size distributions were plotted.

**Hydrodynamic size, PDI, and zeta potential (DLS)**

Hydrodynamic diameter and polydispersity index (PDI) were measured by DLS. Zeta potential was measured using a 1 mg/mL CS-PB dispersion at 25 °C and pH 7.4.

**FT-IR analysis**

For FT-IR analysis, CS-PB powder was mixed with KBr at a 1:100 ratio, ground, and pressed into pellets. Pure KBr was used as the background. Spectra were collected over 4000–400 cm−1 with a resolution of 4.0 cm^-1^ and 32 scans. CS-CND (chitosan–carbon dot composite) was used as a reference control where applicable.

**ESR/EPR analysis**

Free radical scavenging capacity was evaluated by ESR/EPR. Radical-generating systems were prepared with and without CS-PB: (i) a xanthine–xanthine oxidase (XOD) system for generating superoxide radicals (·O2−); and (ii) a Fenton system for generating hydroxyl radicals (·OH). DMPO was used as a spin-trapping agent. ESR parameters were set as follows: center field 3500 G, scan range 3460–3560 G, microwave power 10 mW, modulation frequency 100 kHz, modulation amplitude 1 G, and scan time 40 s.

**Enzyme-Mimetic Activity Assays**

**SOD-like activity assay**

SOD-like activity was measured by a xanthine–XOD–cytochrome c competitive inhibition spectrophotometric method. In a 96-well plate, 50 μL PBS (pH 7.4), 50 μL xanthine solution, and 50 μL cytochrome c solution were added. Ultrapure water and XOD were then added to a final volume of 300 μL. Absorbance change at 550 nm within 1 min was recorded as ΔA1. The volume of XOD was adjusted such that ΔA1 stabilized around 0.025. CS-PB was prepared in eight serially diluted concentrations; 30 μL of nanozyme sample was added per well while keeping other components unchanged, and the absorbance change within 1 min was recorded as ΔA2. Each concentration was tested in triplicate.

The inhibition rate was calculated as:

Inhibition (%) = 0.0225−(ΔA2−ΔA1)/0.0225×100%.

IC50 was obtained by fitting inhibition (%) versus final nanozyme concentration. Specific activity (U/mg) was calculated as:

Specific activity (U/mg) = 1/0.3×IC50.

**Catalase-like activity assay**

Catalase-like activity was evaluated by monitoring dissolved oxygen generation during H_2_O_2_ decomposition. CS-PB (200 μL, 1 mg/mL) was added into 10 mL PBS (pH 7.4), and the baseline dissolved oxygen was recorded (0 min). Then 200 μL of 3% H_2_O_2_ was added, mixed thoroughly, and dissolved oxygen was recorded every 1 min for 10 min. Oxygen generation rate was calculated from the linear region (0–5 min).

**POD-like activity assay**

POD-like activity was assessed using TMB as the substrate in NaAc–HAc buffer (pH 6.8). The 100 μL reaction mixture contained 10 μL CS-PB (1 mg/mL), 5 μL H_2_O_2_, 1 μL TMB stock solution (20 mg/mL in DMSO), and 84 μL buffer. Absorbance at 652 nm was recorded continuously, and the initial rate (ΔA/Δt) within the first 1 min was calculated. Catalytic activity (b) was calculated as:

b=[V/(ε×l)] × [ΔA/Δt],

where V is reaction volume, ε\varepsilonε is the molar extinction coefficient of oxidized TMB (39,000 M^-1^·cm^-1^), and l is the optical path length (0.3 cm).

**OXD-like activity assay**

OXD-like activity was evaluated similarly but without H_2_O_2_. The 100 μL mixture contained 10 μL CS-PB (1 mg/mL), 1 μL TMB stock, and 89 μL NaAc–HAc buffer (pH 6.8). Absorbance at 652 nm was monitored, and the initial ΔA/Δt and specific activity were calculated as described above.

**pH stability of SOD- and CAT-like activities**

To assess pH stability, five pH conditions (4.0, 5.5, 7.4, 8.5, and 10.0) were tested for SOD- and CAT-like activities. SOD-like activity retention was normalized to pH 7.4 as 100%. CAT-like activity retention was calculated based on oxygen generation rates. Each condition was tested in triplicate.

**Supplementary Figures**


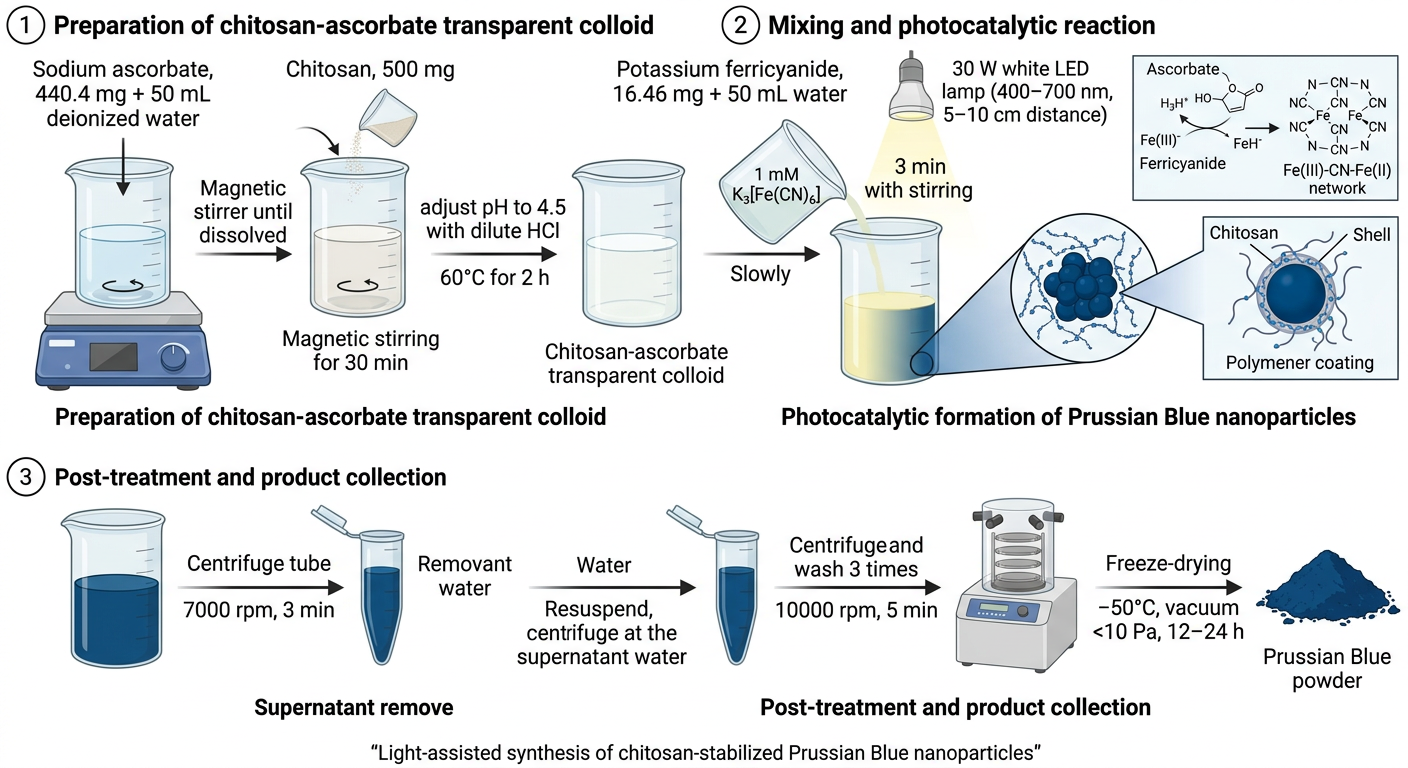


Figure S1. Schematic diagram of CS-PB synthesis.


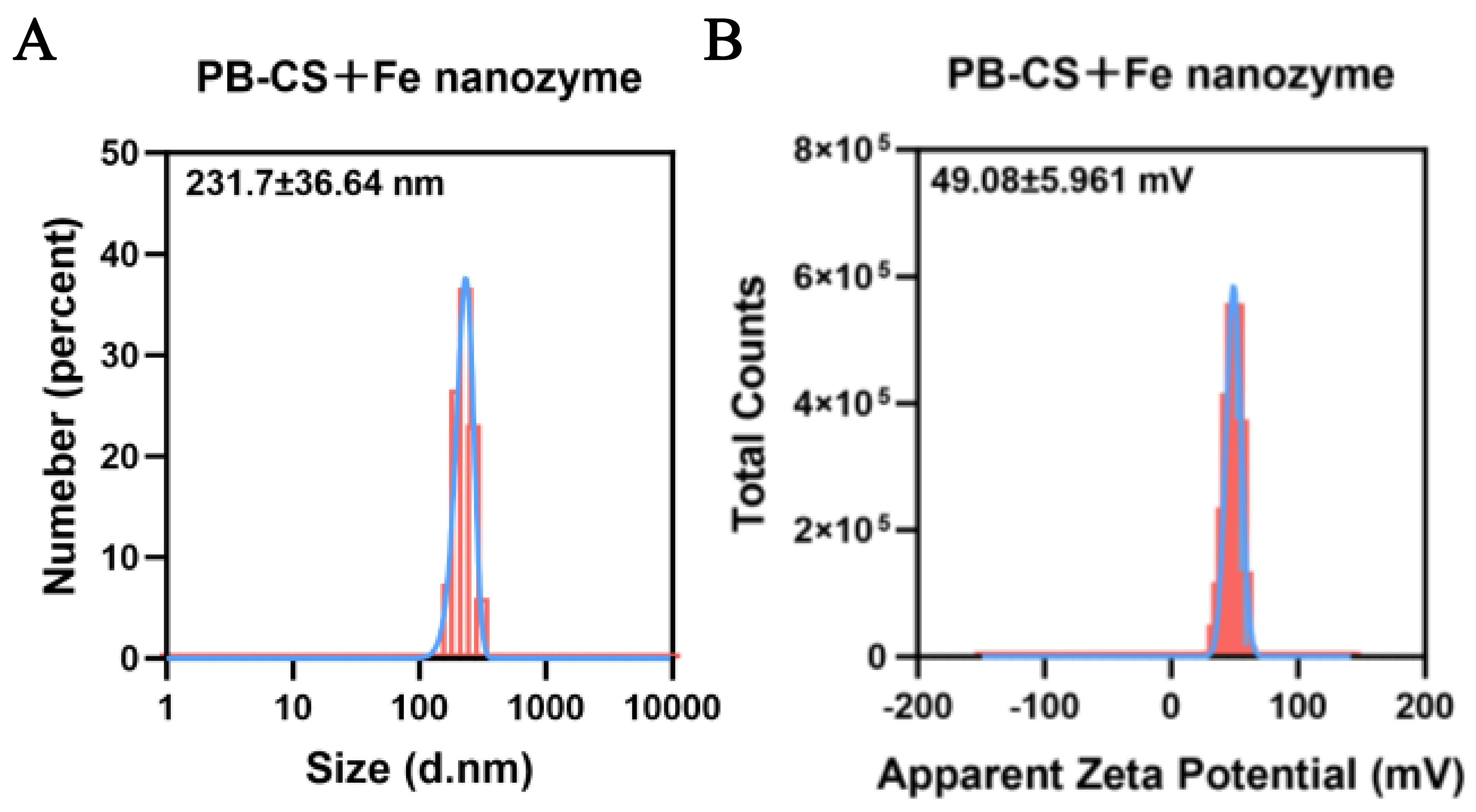


Figure S2. Characterization of CS-PB nanozymes. A. Dynamic light scattering (DLS) particle size distribution of CS-PB nanozymes in aqueous solution, showing a narrow size distribution centered at approximately 42 nm; B. Zeta potential distribution of CS-PB nanozymes, indicating a positively charged surface with an average potential of +18.7 mV, which contributes to the colloidal stability of the nanoparticles.


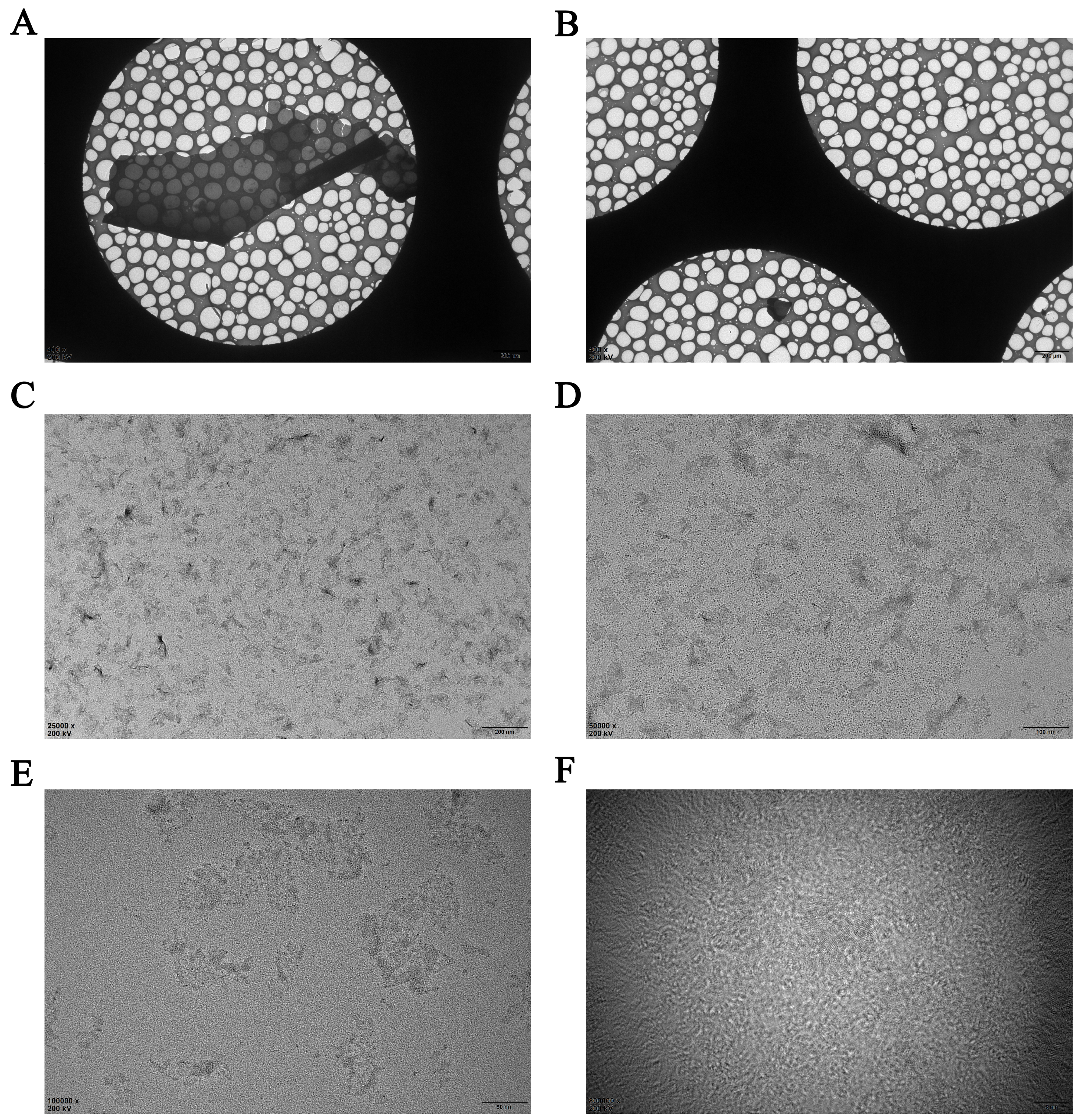


Figure S3. TEM and HRTEM characterization of CS-PB nanozyme. A, B. Low-magnification TEM images showing the overall morphology and dispersion of CS-PB nanoparticles; C–E. High-magnification TEM images revealing nanoscale particle distribution; F. HRTEM image showing lattice fringes, confirming crystalline characteristics of the Prussian blue component.


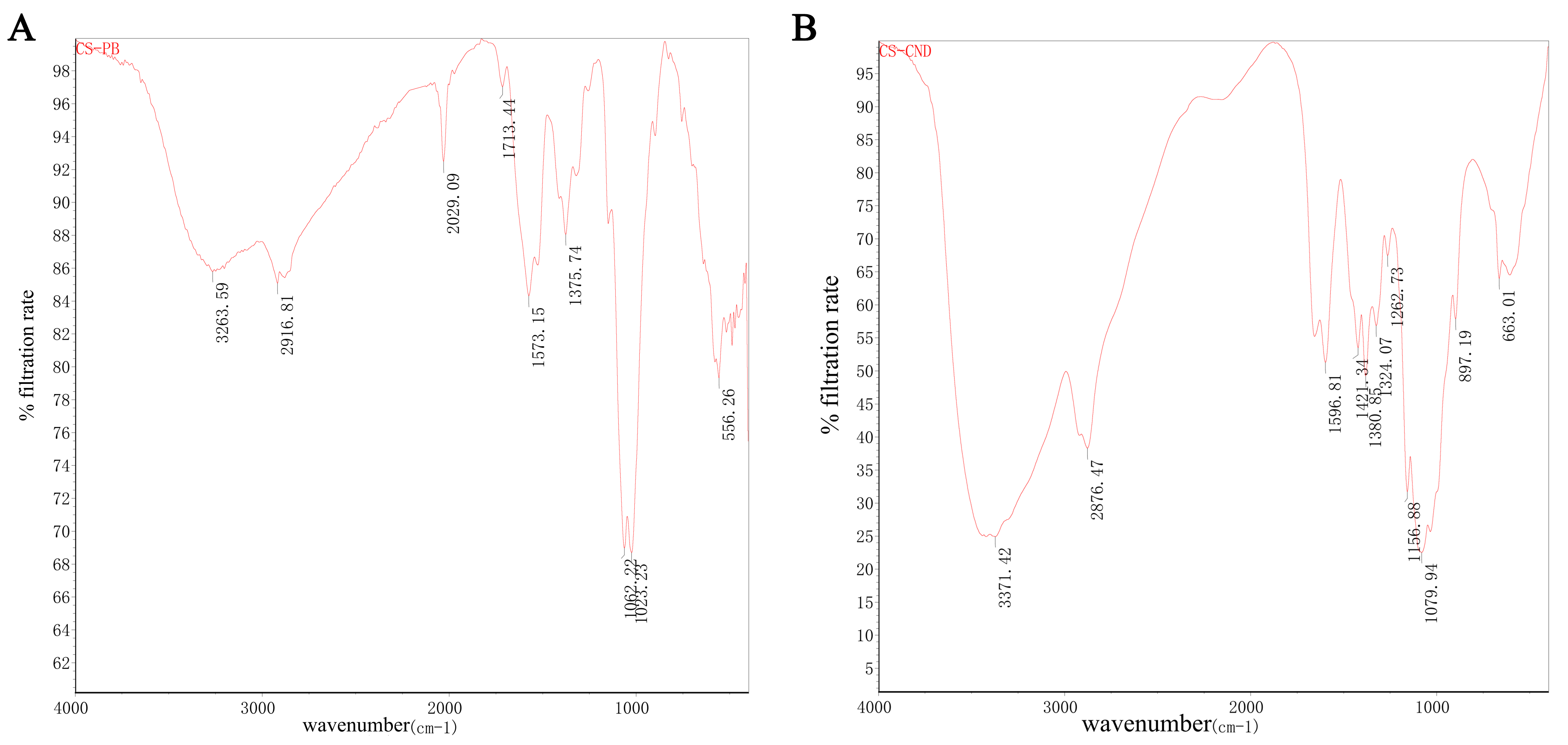


Figure S4. FT-IR testing. A. FT-IR spectrum of CS-PB nanozyme in the range of 4000–500 cm^-1^, with labeled characteristic absorption peaks; B. FT-IR spectrum of CS-CND control sample in the same wavenumber range, with labeled characteristic absorption peaks. Both spectra were acquired under identical instrumental conditions (32 scans, 4 cm^-1^ resolution, DTGS KBr detector).


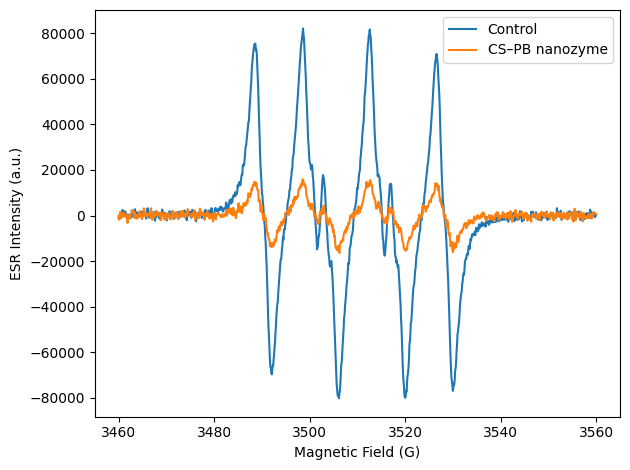


Figure S5. ESR spectra of the control group and CS-PB nanozyme group in the magnetic field range of 3460–3560 G, reflecting the reactive oxygen species (ROS) radical signal intensity of the two groups.


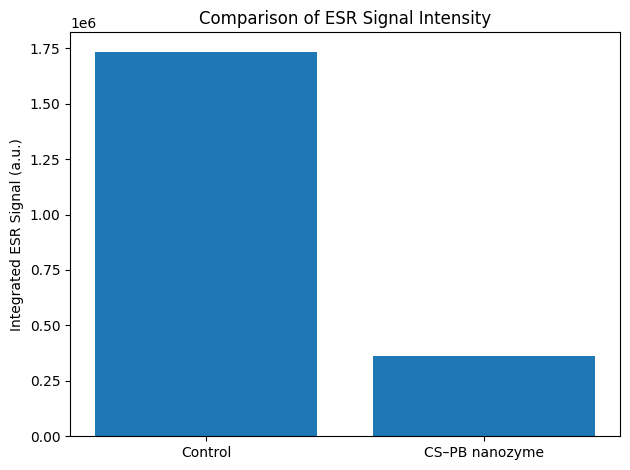


Figure S6. Comparison of integrated ESR signal intensity between the control group and CS-PB nanozyme group, quantifying the ROS-scavenging capacity of the nanozyme.


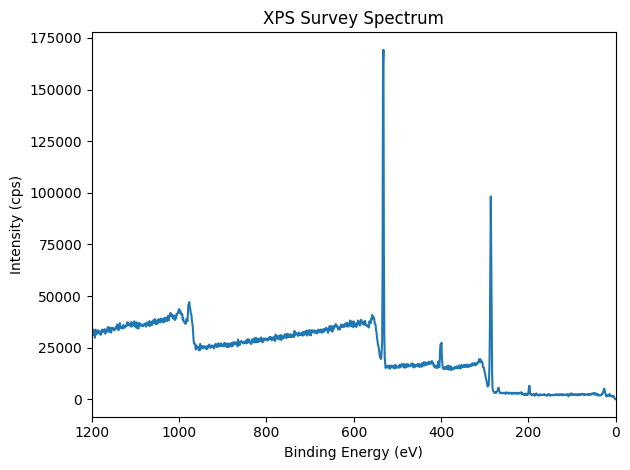


Figure S7. XPS survey spectrum of CS-PB nanozyme, showing the surface elemental composition (carbon, oxygen, iron) and corresponding binding energy peaks of the sample.


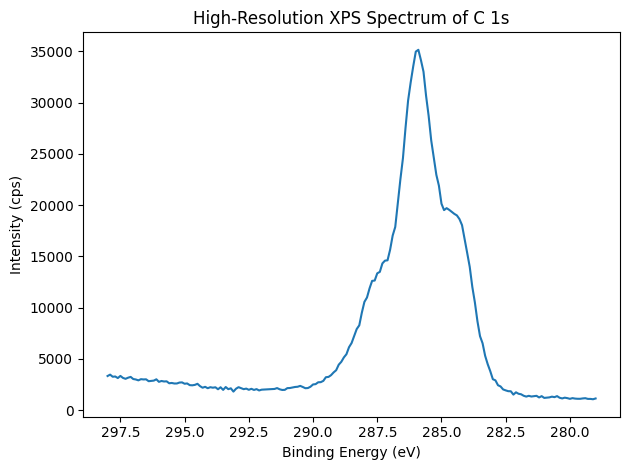


Figure S8. High-Resolution XPS Spectrum of C 1s for CS-PB nanozyme, displaying the characteristic peak of C–C/C–H bonds and the shoulder peaks of oxygen- and nitrogen-containing functionalities in the chitosan backbone.


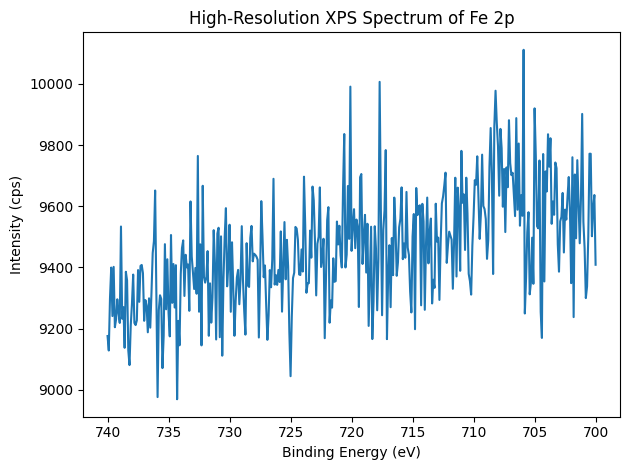


Figure S9. High-resolution XPS Spectrum of Fe 2p for CS-PB nanozyme, showing the discernible Fe-related signal peaks of the sample.


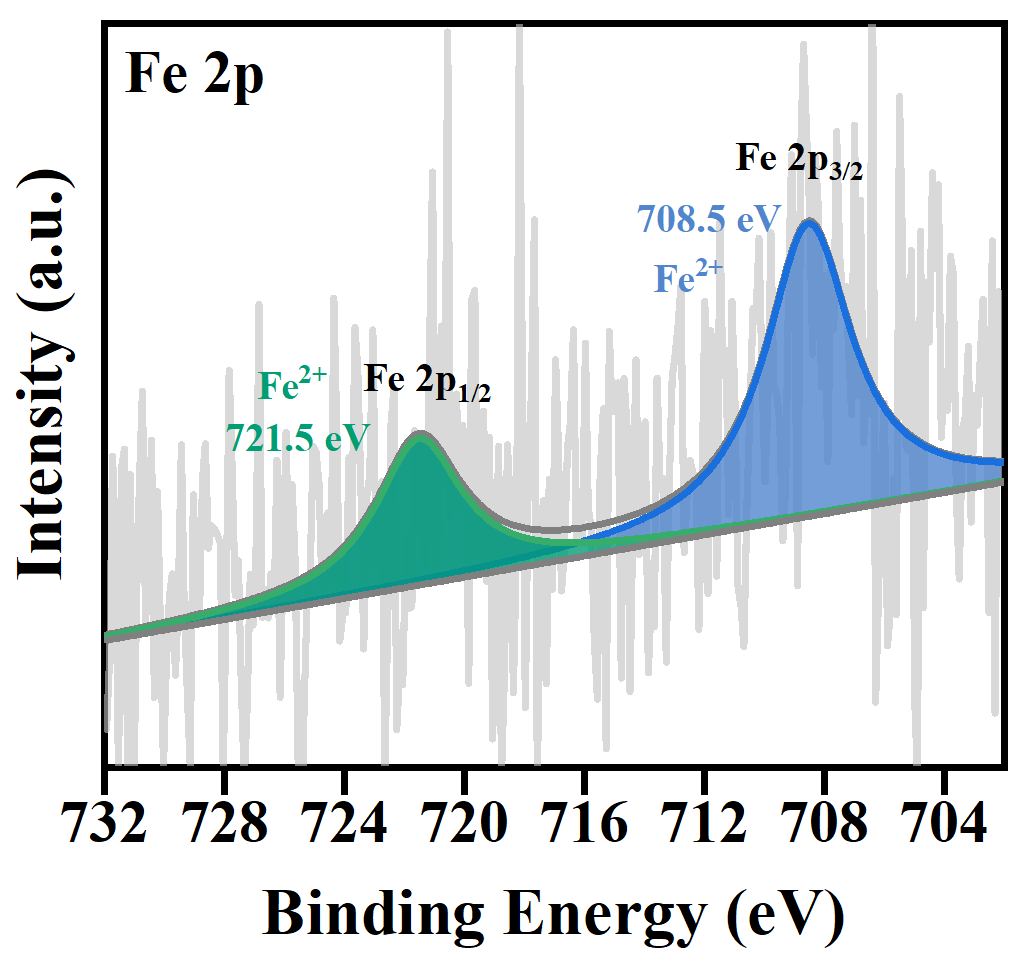


Figure S10. High-resolution XPS analysis spectrum of Fe 2p for CS-PB nanozyme, presenting the characteristic peaks of Fe 2p3/2 and Fe 2p1/2 and the corresponding binding energies.


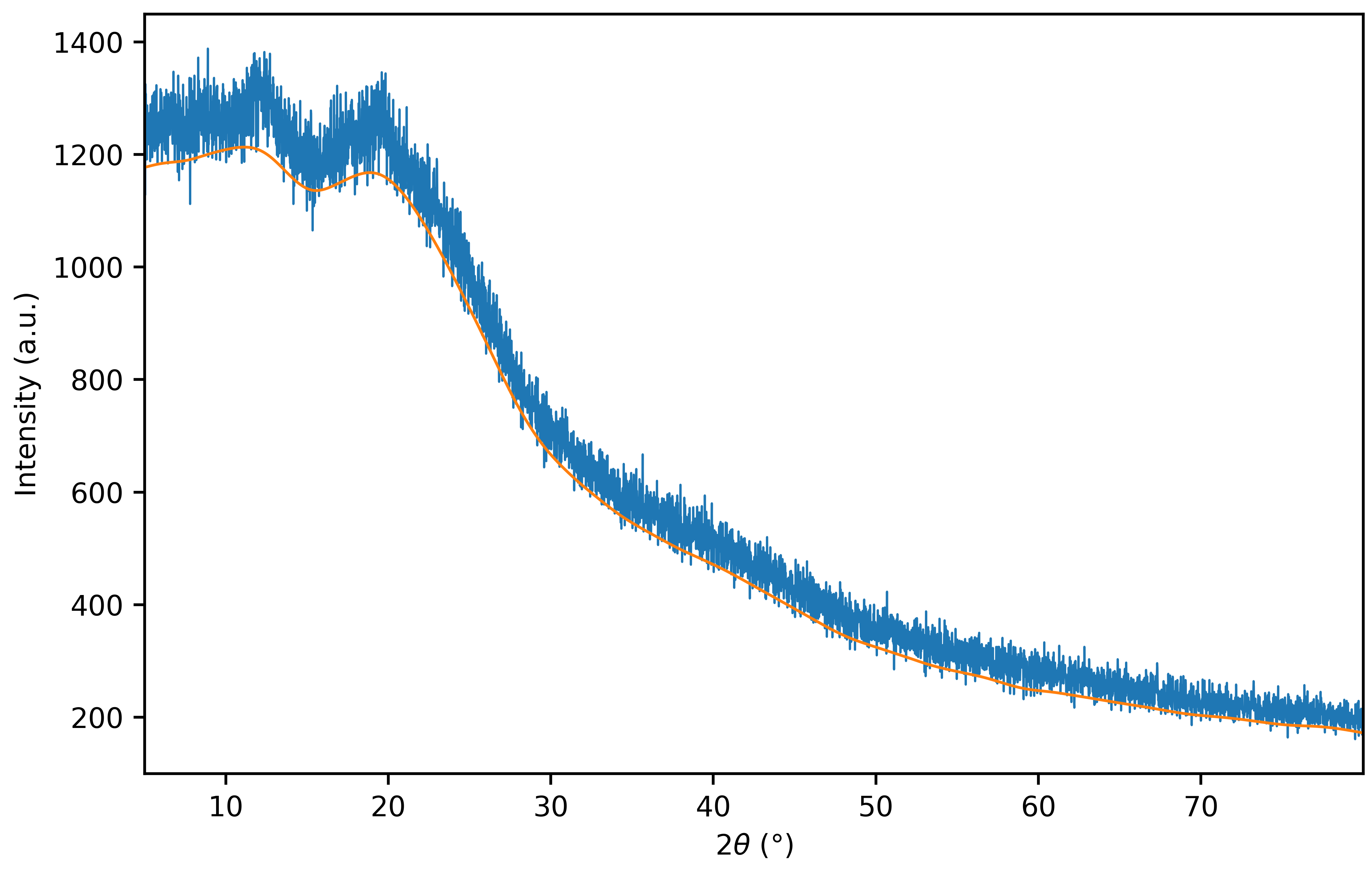


Figure S11. XRD pattern of CS-PB nanozyme within the 2θ scanning range of 5–80°, showing the original diffraction peaks of the material.


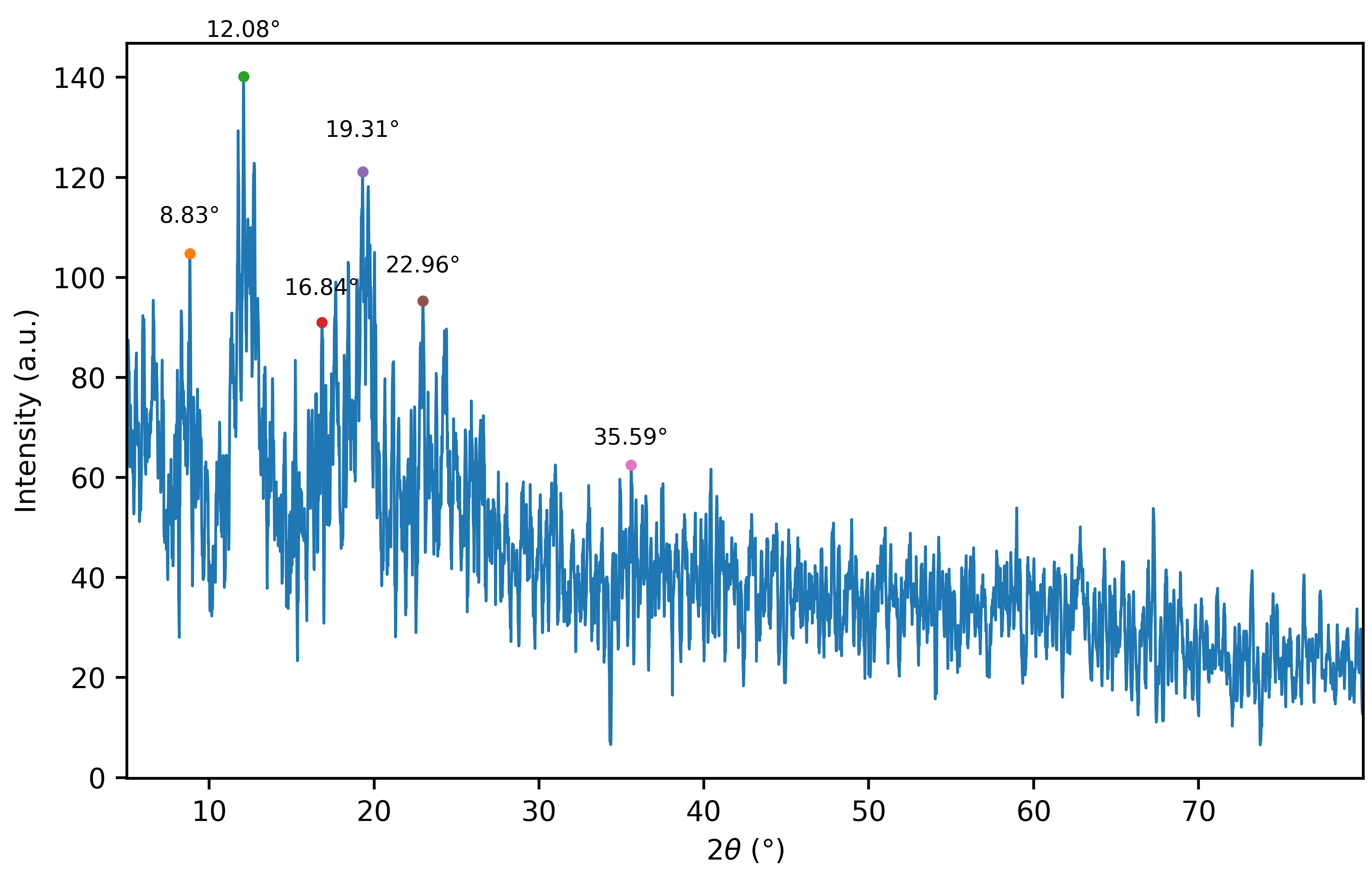


Figure S12. Background-subtracted and smoothed XRD pattern of CS-PB nanozyme, marking the major and minor characteristic diffraction peaks at different 2θ angles.


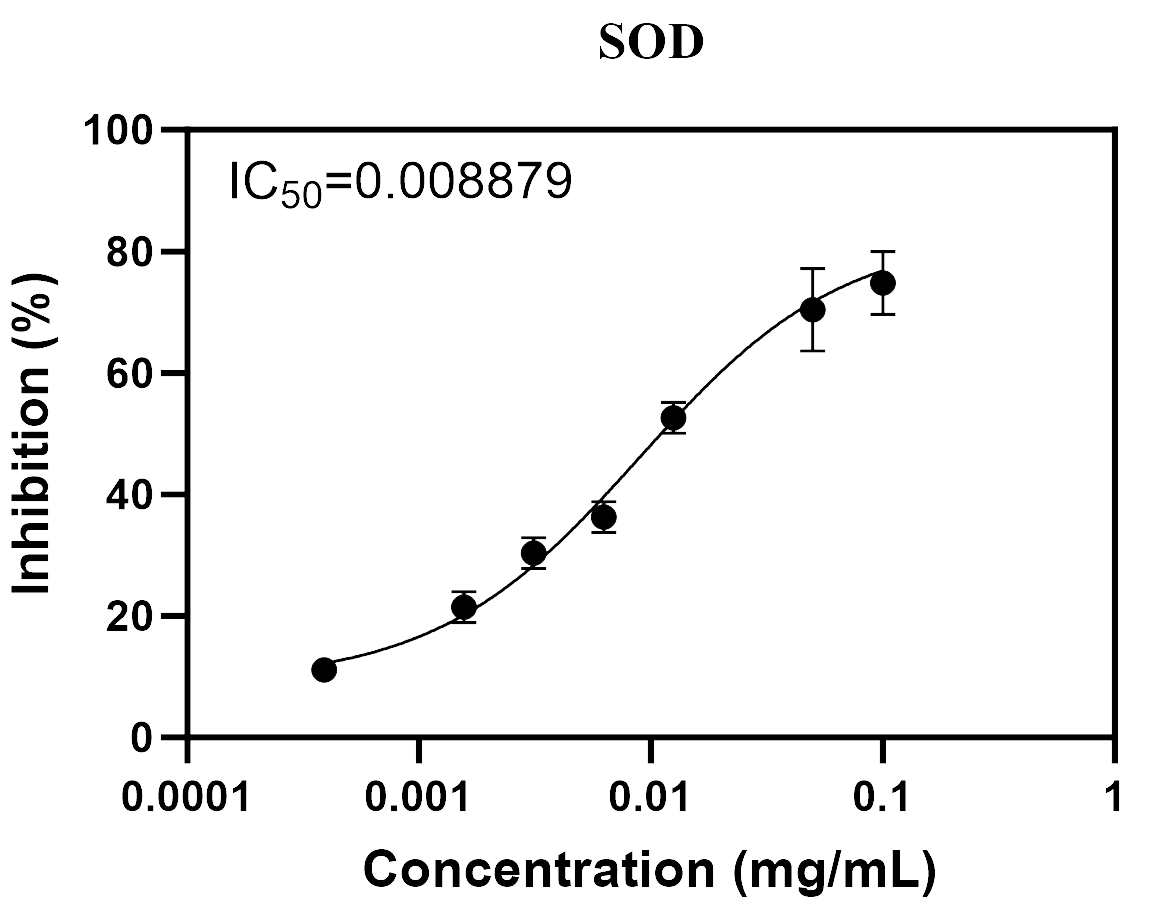


Figure S13. SOD-like activity inhibition curve of CS-PB nanozyme, showing the concentration-dependent inhibition rate of superoxide anion radicals and the calculated IC50 value.


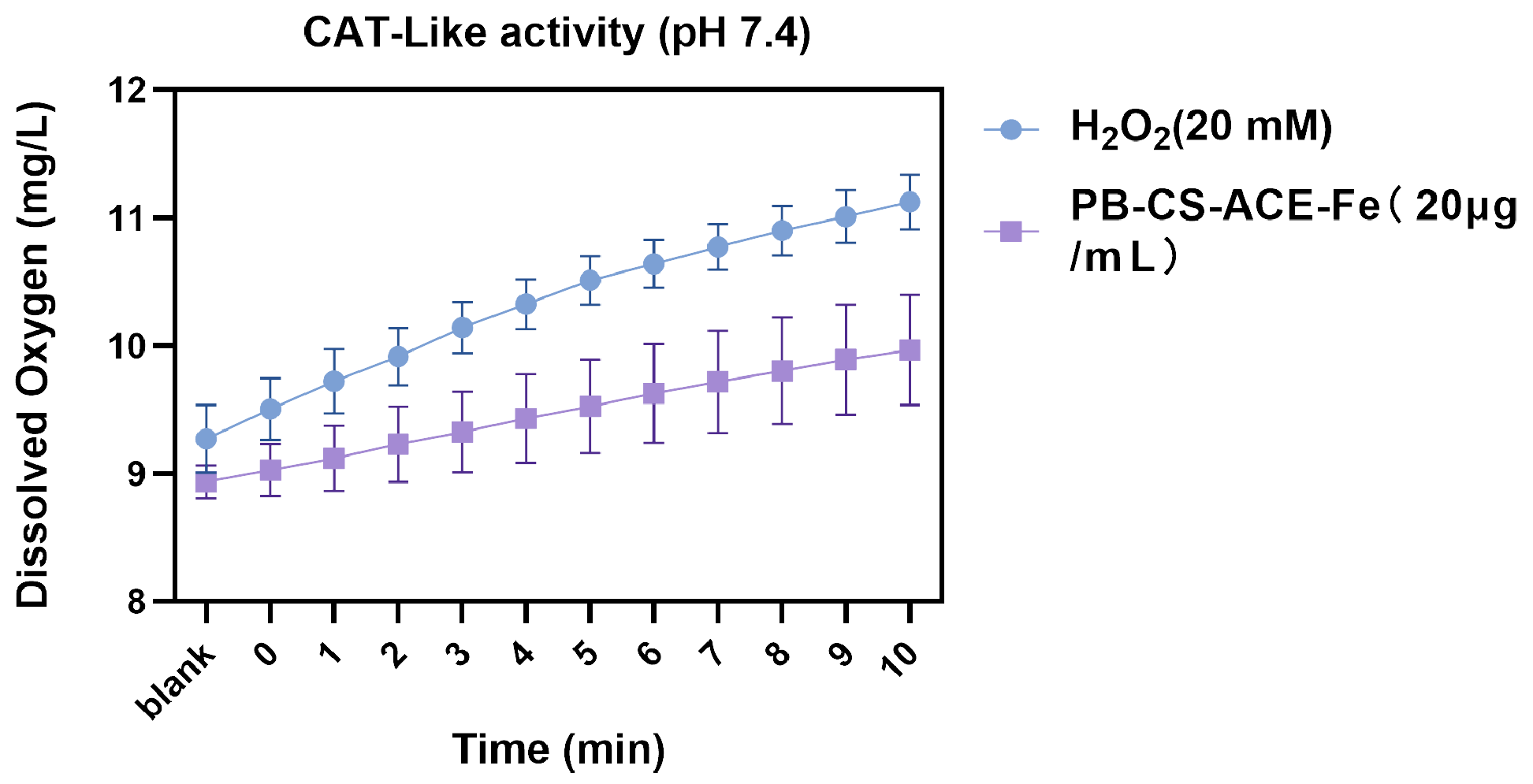


Figure S14. Dissolved oxygen change curve of CS-PB nanozyme during H_2_O_2_ decomposition, reflecting the CAT-like activity and the linear oxygen generation rate in the initial 5 min.


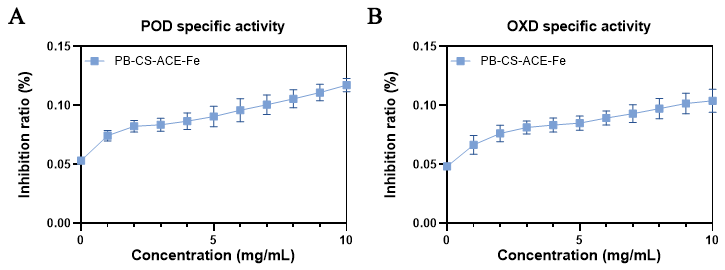


Figure S15. POD-like and OXD-like specific activity of PB-CS-ACE-Fe at different concentrations, showing the concentration-dependent catalytic activity of the nanozyme for TMB oxidation. A. POD-like; B. OXD-like.


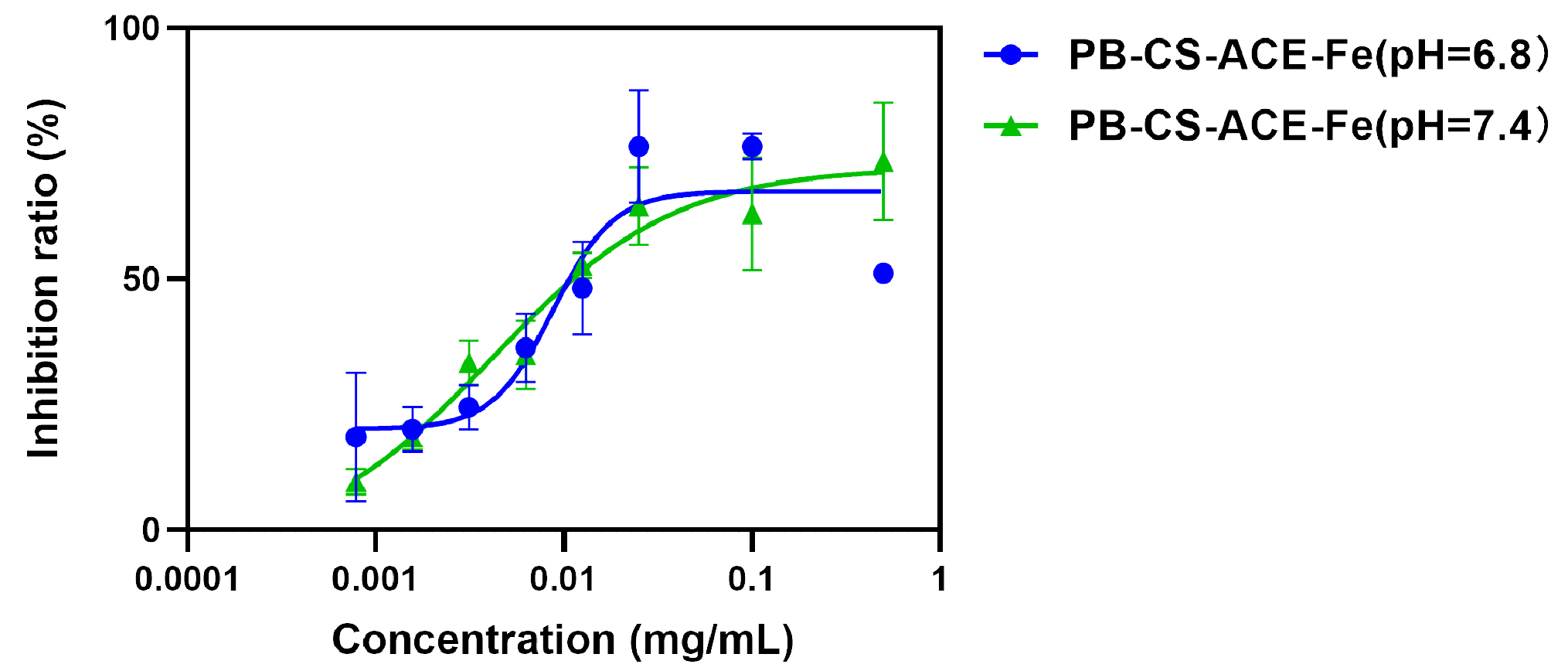


Figure S16. SOD-like activity inhibition rate of CS-PB nanozyme at pH 6.8 and pH 7.4, comparing the catalytic activity of the nanozyme under mildly acidic and physiological pH conditions.


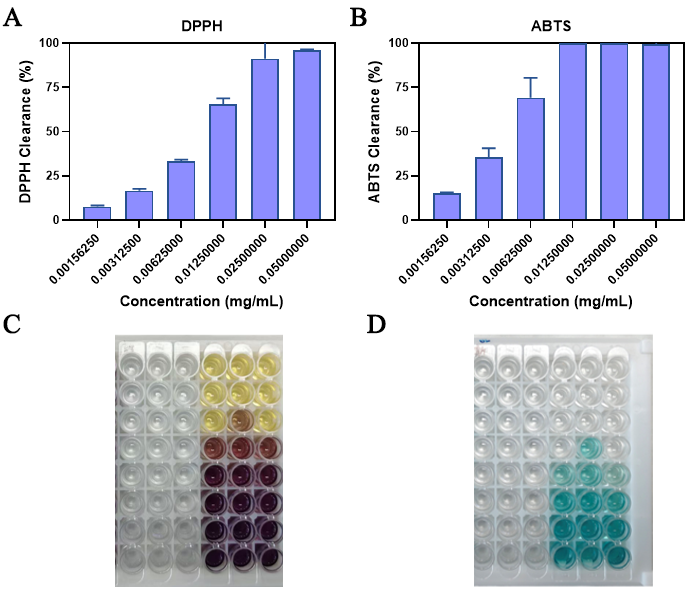


Figure S17. Antioxidant activity of PB-CS-ACE-Fe. A. DPPH radical scavenging activity of PB-CS-ACE-Fe at various concentrations (0.0015625–0.05 mg/mL); A. ABTS radical scavenging activity of PB-CS-ACE-Fe at various concentrations (0.0015625–0.05 mg/mL); C, D. Corresponding 96-well plate photographs showing the colorimetric changes of (C) DPPH and (D) ABTS solutions after reaction with PB-CS-ACE-Fe.


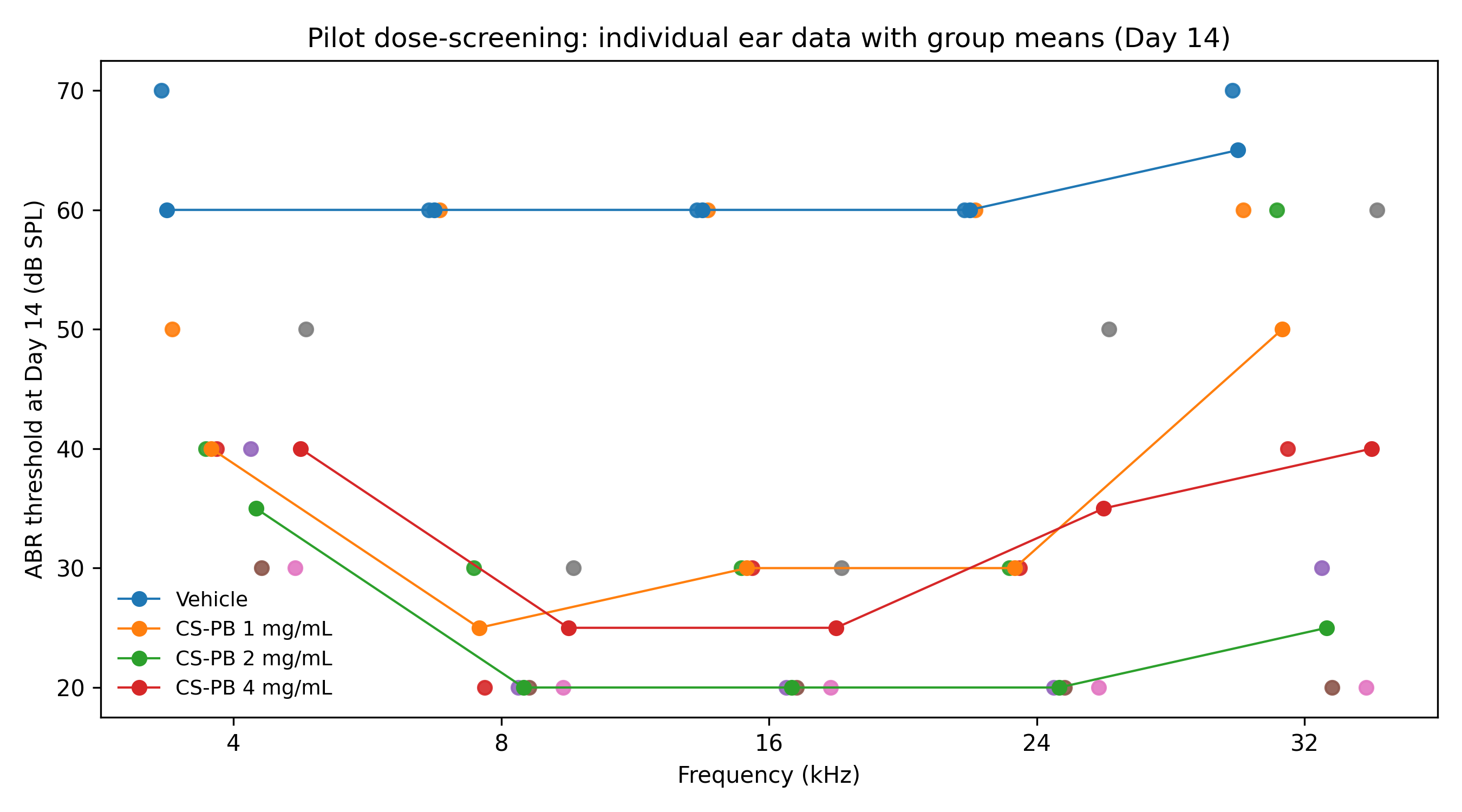


Figure S18. ABR thresholds (dB SPL) at different frequencies on Day 14 post-noise exposure across experimental groups. ABR, auditory brainstem response; CS-PB, chitosan-Prussian blue nanozyme.

**Supplementary Table**

Table S1. Raw ABR thresholds of individual ears at Day 14.

| Group | Ear | 4 kHz | 8 kHz | 16 kHz | 24 kHz | 32 kHz |
| --- | --- | --- | --- | --- | --- | --- |
| Control | Ear1 | 70 | 60 | 60 | 60 | 70 |
| Control | Ear2 | 50 | 60 | 60 | 60 | 60 |
| 1 mg/mL | Ear1 | 40 | 20 | 30 | 30 | 40 |
| 1 mg/mL | Ear2 | 40 | 30 | 30 | 30 | 60 |
| 2 mg/mL | Ear1 | 40 | 20 | 20 | 20 | 30 |
| 2 mg/mL | Ear2 | 30 | 20 | 20 | 20 | 20 |
| 4 mg/mL | Ear1 | 30 | 20 | 20 | 20 | 20 |
| 4 mg/mL | Ear2 | 50 | 30 | 30 | 50 | 60 |
